# Sickness absence trajectories among young and early midlife employees with psychological distress: the contributions of social and health-related factors in a longitudinal register linkage study

**DOI:** 10.1007/s00420-024-02114-7

**Published:** 2024-12-05

**Authors:** Jatta Salmela, Noora Amanda Heinonen, Jade Knop, Marianna Virtanen, Pi Fagerlund, Anne Kouvonen, Tea Lallukka

**Affiliations:** 1 Department of Public Health, University of Helsinki, Helsinki, Finland; 2 Faculty of Social Sciences, University of Helsinki, Helsinki, Finland; 3 Department of Educational Sciences and Psychology, University of Eastern Finland, Kuopio, Finland; 4 Division of Insurance Medicine, Department of Clinical Neuroscience, Karolinska Institute, Stockholm, Sweden; 5 Centre for Public Health, Queen’s University Belfast, Belfast, Northern Ireland

**Keywords:** Employee cohort, Occupational health, Psychological distress, Sickness absence, Trajectory modelling

## Abstract

**Purpose:**

Psychological distress has been associated with sickness absence (SA), but less is known about whether there are distinct patterns in the development of SA among people with psychological distress. We examined trajectories of short- and long-term SA among employees with psychological distress and how social and health-related factors are associated with them.

**Methods:**

We used the employer’s register data on all-cause short- (≤ 10 working days) and long-term (> 10 working days) SA with a two-year follow-up. We prospectively linked the Helsinki Health Study survey data on 19–39-year-old employees of the City of Helsinki, Finland, in 2017, to the SA data. We included 1060 participants (81% women) who reported experiencing psychological distress, measured by the emotional wellbeing scale of RAND-36. Survey responses of age; gender; education; marital status; social support, procedural and interactional organisational justice, and bullying at work; physical activity; diet; tobacco and alcohol use; prior SA; and the level of psychological distress were included as exposures. Group-based trajectory modelling and multinomial logistic regression were used for the analyses.

**Results:**

We identified four short-term SA trajectories: ‘low’ (n = 379, 36% of participants), ‘descending’ (n = 212, 20%), ‘intermediate’ (n = 312, 29%), and ‘high’ (n = 157, 15%); and two long-term SA trajectories: ‘low’ (n = 973, 92%) and ‘high’ (n = 87, 8%). A higher education, fewer prior SA, and lower levels of psychological distress were associated with the ‘low’ short- and long-term SA trajectories.

**Conclusion:**

SA trajectories differ among employees with psychological distress. Early intervention and support are needed among employees with mental health symptoms to prevent future SA.

**Supplementary Information:**

The online version contains supplementary material available at 10.1007/s00420-024-02114-7.

## Introduction

Over the last couple of decades, several studies have reported increasing psychological distress across developed countries, especially in young and early midlife adults (Gagné et al. 2022; Knapstad et al. 2021; McGinty et al. 2020). The COVID-19 pandemic further increased the prevalence of psychological distress, although some of its impacts have diminished thereafter, and even some positive trends have been observed (Daly and Robinson 2020; Gagné et al. 2022; Reutter et al. 2024). Psychological distress is a commonly used indicator of population mental health (Drapeau et al. 2012) and a measure of self-reported psychological problems (Bültmann et al. 2005). It is characterised by emotional burden or suffering often experienced in common mental disorders, such as depressive and anxiety disorders (Mirowsky and Ross 2002; Goodwin et al. 2013). Given the high prevalence, mental health problems cause a remarkable human and economic burden. They have been associated with several chronic diseases and comorbidity, and have a high recurrence (Jacobi et al. 2004; Kessler et al. 2007; Prince et al. 2007).

Additionally, psychological distress has been associated with a higher risk of sickness absence (SA) (Bültmann et al. 2005; Virtanen et al. 2007; Halonen et al. 2020). The risk also applies to sub-clinical levels of psychological symptoms, indicating everyday ‘stress of life’ (Terluin et al. 2011). Among midlife Finnish employees, greater severity of common mental disorders was associated with a higher risk of SA spells of different lengths (Mauramo et al. 2018). SA spells cause a considerable burden for societies, employers, and individuals due to their direct and indirect economic costs (e.g., salary and benefit costs and decreased productivity) and because of their social consequences (e.g., isolation and loneliness) (Hultin et al. 2012; Knapstad et al. 2014). These economic and social burdens may further deteriorate individuals’ mental health (Knapstad et al. 2014).

As mental health problems have been shown to increase especially in young employees, it is of importance to explore whether and how they contribute to SA. Mental health symptoms do not necessarily lead to SA since other factors, such as the work environment, influence work participation (Ervasti et al. 2017). It is likely that individuals with psychological distress are not a homogeneous group with similar patterns of future SA. For example, higher age, comorbidity, and weak self-esteem have predicted disability pension among workers with clinical depression (Rytsälä et al. 2007). Additionally, a previous study of midlife employees found that the predictors of work disability trajectories were largely, though not fully, similar between those with and without common mental disorders (Hiilamo et al. 2019). However, to the best of our knowledge, no prior research has examined the possible heterogeneity of SA patterns specifically among employees with psychological distress. Most of the previous research has focused on general population groups (including individuals with and without symptoms), used variable-oriented methods instead of person-oriented methods, or ignored the varying lengths of SA spells. Thus, more information is needed about the distinct pathways of SA among people with psychological distress, for instance, for the purposes of secondary prevention. To fill this knowledge gap, this study aims to identify SA trajectories among young and early midlife employees with psychological distress.

In the current study, we examine trajectories of short- (≤ 10 working days) and long-term (> 10 working days) SA separately, as it is possible that short-term SA spells are to some extent determined by different factors than long-term SA. Furthermore, while long-term SA requires a medical diagnosis, reasons for short-term—that is, self-certified—SA remain unclear (Hultin et al. 2012). Generally, long-term SA has been associated with more severe health problems, such as chronic diseases, which increase with age, hamper work ability (Marmot et al. 1995; Sumanen et al. 2015), and are influenced by genetics and lifestyle factors (Leineweber et al. 2020). In contrast, short-term SA spells have been related to common and less severe health problems, such as respiratory diseases and headache (Feeney et al. 1998; Szubert et al. 2016), and also lifestyle factors, such as smoking and physical inactivity (Kanerva et al. 2018; Salmela et al. 2023). Moreover, there is some evidence that short-term SA would be especially related to work (Leineweber et al. 2020), as it could be used for coping at work (de Boer et al. 2002), indicating an employee’s poor possibilities for adjusting their work (Leineweber et al. 2020). However, more evidence is needed in younger employees, who have been found to have more short-term SA than older workers (Sumanen et al. 2015).

In addition, we investigate social and health-related factors, such as psychosocial working conditions and lifestyles, as possible modifiable factors that might be associated with SA trajectories. For example, job demands and job control (Amiri and Behnezhad 2020), workplace bullying (Løvvik et al. 2022; Nielsen et al. 2016), and injustice at the workplace (Elovainio and Kivimäki 2002; Ybema and van den Bos 2010) have been associated with a higher likelihood of subsequent SA. Low physical activity, an unhealthy diet, smoking, and binge drinking have also been associated with future SA (Lahti et al. 2012; Virtanen et al. 2018; De Bortoli et al. 2021; Salmela et al. 2023). Prior research suggests that interventions that target both the management of risk factors in the work environment and the promotion of healthy lifestyles are the most beneficial in the reduction and prevention of SA (Anger et al. 2015; Feltner et al. 2016).

More information on the protective and risk factors for SA in those with psychological distress is important from the preventive point of view, as recurring short-term SA spells have been associated with subsequent long-term SA due to mental disorders among young employees (Harkko et al. 2021; Sumanen et al. 2017). Furthermore, long-term SA has been shown to predict future disability pension (Helgesson et al. 2015; Klein et al. 2021). By examining different lengths of SA in those with psychological distress, this study provides information that can be used to develop secondary prevention interventions. Prevention of short- and long-term SA is important not only from the individuals’ perspective but also from the perspective of reducing the costs caused by sickness allowances. This applies to costs to employers, because in Finland as well as in many other countries, short-term SA is typically compensated by employers.

## Methods

### Data and population

The data were derived from the Helsinki Health Study, a longitudinal cohort study focusing on the social and behavioural determinants of health and wellbeing among municipal employees. This study focused on employees of the City of Helsinki aged 19–39 years in 2017, representing a large number of occupations in different sectors, the largest sectors being health and social care and education. The original sample consisted of 5898 participants with a response rate of 51% (Lallukka et al. 2020). The majority of the participants (81%) were women, corresponding to the gender distribution in the public sector in Finland in general (Lallukka et al. 2020). In this study, we derived our study sample among the participants who gave their informed consent to register-linkage (n = 4864).

**Fig. 1:**
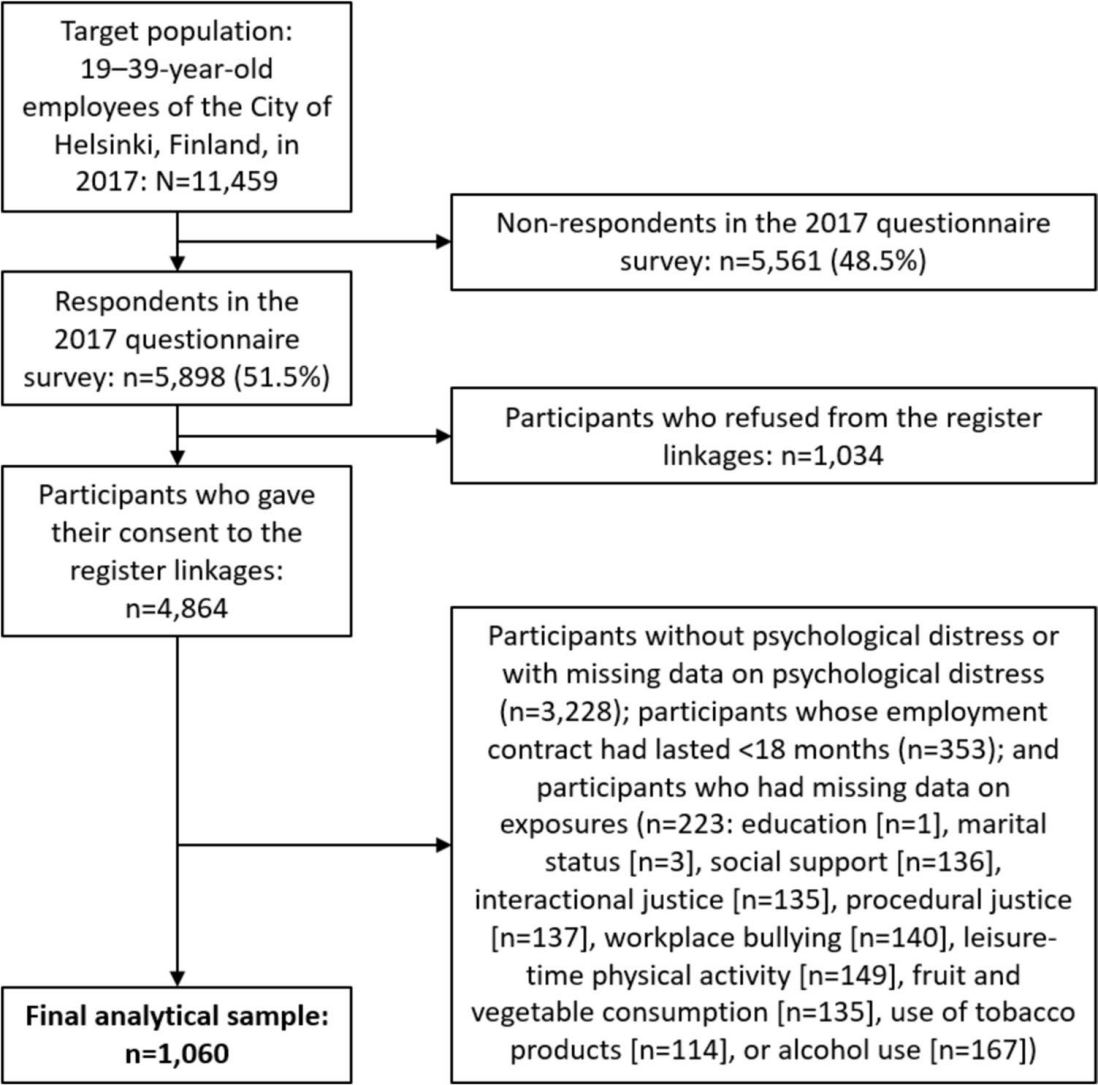
Flow diagram of the selection of the final study population among the Helsinki Health Study participants in 2017 (n = 1060)

We included participants who reported psychological distress in 2017, defined by the RAND-36 emotional wellbeing subscale, which is a validated instrument for assessing health-related quality of life (Hays and Morales 2001). The instrument consists of five questions where the participants are asked to evaluate the frequency of different emotional states over the past four weeks: “Have you been a very nervous person?”, “Have you felt so down in the dumps that nothing could cheer you up?”, “Have you felt calm and peaceful?”, “Have you felt downhearted and blue?”, and “Have you been a happy person?”. A summary score ranging between 0 and 100 was calculated for each participant, lower values indicating more symptoms of distress. We divided participants into tertiles and selected the lowest tertile—the cut-off being 68/100—to indicate psychological distress. This corresponds to Yamazaki’s et al. (2005) study where a cut-off score of 68/100 was used to indicate mild, moderate, or severe depressive symptoms. It is also in line with Cuijpers’ et al. (2009) study where a cut-off score of 74/100 was used for major depression, and 62/100 for generalised anxiety disorder, and with Ten Haven’s et al. (2024) study where the cut-off scores of 68/100 and 76/100 were found to have the highest sensitivity and specificity for mood disorders and anxiety disorders, respectively. Consequently, we excluded participants with scores over 68 (i.e., no psychological distress; n = 3213), and also those with missing values (n = 15). Furthermore, we excluded the participants whose employment had lasted under 18 months (n = 353) to ensure long enough SA follow-up time (at least 3 out of 4 measurement points) for all participants, and who had missing data on exposure variables (n = 223). A total of 1,060 participants met the inclusion criteria and were included in the final data analysis (Fig. 1).

### Outcome

#### Sickness absence

Data on SA were derived from the personnel register of the City of Helsinki. The follow-up began one day after returning the survey and ended on the 31st of March 2020 or on the last day of the employment contract, whichever occurred first. The date was chosen to exclude the influence of the COVID-19 pandemic on SA. In Finland, short-term SA spells lasting the maximum of 10 working days are covered by the employer whereas long-term SA spells exceeding 10 working days but lasting a maximum of 300 days during a 2-year period are fully covered by the Social Insurance Institution of Finland and require a medical certificate (Kela 2024). In accordance with this, we classified the SA spells as short-term (≤ 10 working days) or long-term (> 10 working days), as done in previous Finnish studies (Fagerlund et al. 2023; Harkko et al. 2021) but also in some other studies from Nordic countries (Nielsen et al. 2023).

We measured short-term SA by the total number of days of short-term SA within each six-month period during the follow-up. Because there were only a fairly small number of long-term SA spells in our data, we measured long-term SA dichotomously by having or not having a long-term SA spell during each six-month period of the follow-up. The mean follow-up period was 2.4 years (range 1.5–2.5 years). As most of the participants had at least two years of follow-up, we restricted the follow-up to two years (i.e., four six-month periods). Only 77 participants had a follow-up of under 2 years.

### Exposures

#### Social factors

We derived social factors from the Helsinki Health Study questionnaire survey. We categorised age as follows: 20–29, 30–34, and 35–39 years, and used gender as a binary variable (woman / man). Education was measured by the highest educational attainment with six response alternatives, and we categorised it into three hierarchical classes: master’s degree or higher, bachelor’s degree, and upper secondary school/vocational education or lower. Marital status was inquired with five response alternatives, and we dichotomised it into ‘married, cohabiting, or in a registered partnership’ and ‘without a partner’ (never married, divorced, or widowed).

Social support at work was measured with four items on help and support received from a supervisor and colleagues (Sarason et al. 1983), including four response alternatives: ‘often’, ‘sometimes’, ‘seldom’, and ‘never’. We summed up the items for a score and allowed one missing answer on the items. We divided the score into tertiles, which yielded the following categories: low, average, and high social support (Laine et al. 2014). Interactional and procedural justice at work were assessed with four items each, including five response alternatives ranging from ‘fully agree’ to ‘fully disagree’ (Moorman 1991). Interactional justice refers to the fairness of the supervisor’s behaviour towards employees, and procedural justice to the fairness of the decision-making process at the workplace. We allowed participants to have one missing answer per scale. We divided both scores into tertiles, which yielded the following categories: low, average, and high level of justice (Koskenvuori et al. 2021). Workplace bullying was measured by the following question: “Mental violence or workplace bullying means isolation of a member of the organisation, underestimation of work performance, threatening, talking behind one’s back or other pressurizing. Have you been subjected to such bullying behaviours?”. Response alternatives were ‘currently’, ‘previously at this workplace’, ‘previously at another workplace’, ‘never’, and ‘I do not know’, and we combined the two response alternatives describing previous bullying (Lallukka et al. 2012).

#### Health-related factors

We derived health-related factors—except prior SA—from the survey. Leisure-time physical activity (LTPA) was assessed with four items where participants estimated their number of weekly hours of LTPA at four levels of exertion. The estimates were transformed into metabolic equivalent (MET) hours per week, which we classified into three groups: low (MET < 14), moderate (MET 14–30), and high (MET > 30) LTPA (Lahti et al. 2010). Fruit and vegetable (F&V) consumption was measured with 2 items from a 14-item food frequency questionnaire. We classified participants into those consuming F&V at least once a day and those eating F&V less often (Salmela et al. 2023). Participants were inquired about their use of tobacco products, and we merged those using tobacco, snus, or electronic cigarettes daily or occasionally into one group, and those not using these products currently or ever into the other (Salmela et al. 2023). Alcohol use was measured with three items on the weekly intake of beer, wine, and spirits, and one item on the frequency of binge drinking. We defined binge drinking as drinking six or more alcohol units per occasion. One unit is equivalent to a bottle of beer or a glass of wine (12 g of pure alcohol). We classified participants into having high alcohol use if their weekly alcohol use was more than 7 units for women and more than 14 units for men, and if binge drinking was more frequent than once a month (Salonsalmi et al. 2009; Salmela et al. 2023). We classified the rest as having moderate alcohol use, including not using alcohol at all.

We derived data on prior SA from the employer’s register and used the total number of SA days over 12 months prior participant’s response to the survey. We classified prior SA into tertiles: 0–3, 4–11, and over 11 days, and those who were employed less than one year (not comparable data on SA for the analysis). In this study, the participants consisted of those who reported psychological distress, defined by the cut-off of score of 68/100 in the emotional wellbeing subscale of RAND-36. However, the participants had varying degrees of psychological distress, and thus, we examined whether more severe psychological distress was associated with the SA trajectories. We then used the cut-off score of 50/100 to dichotomise participants into those with high (score 0–50) and those with moderate (score 50–68) psychological distress. This corresponds to the cut-off score used for severe depressive symptoms (52/100) in Yamazaki et al.’s (2005) study and for probable depressive disorder (48/100) in Silveira’s et al.’s (2005) study.

### Statistical analyses

Trajectories of short- and long-term SA were examined using group-based trajectory modelling (GBTM), which is an application of finite mixture modelling that identifies groups of individuals following similar trajectories of an outcome over time (Nagin 2014). The identified number of latent trajectories, including their shapes and group sizes, are based on their interpretability and designated statistical criteria (Nagin and Odgers 2010). In this study, we modelled the number of days of short-term SA per six months with a zero-inflated Poisson distribution, as there was an excess of participants with zero SA spells in the data. For long-term SA, we used the binary logistic distribution in the models, and thus, estimated the probabilities of having long-term SA spells per six months. We started the model selections by fitting a model with one zero-order (i.e., intercept) trajectory and one quartic trajectory (Tables S1 and S2). We increased the number of quartic trajectories until there was no improvement in the Bayesian Information Criterion (BIC) value and as long as the size of each group was at least 5%. After establishing the number of groups, we decreased the polynomial order of each trajectory until the highest polynomial was significant at level α = 0.05 or as long as there was improvement in the BIC value. We assessed the adequacy of the selected model based on the average posterior probabilities (APP) (minimum threshold of 0.7), the odds of correct classification (OCC) (minimum threshold of 5), entropy (values closer to 1 indicate better fit), and the correspondence between the assigned proportions and the estimated probabilities of each group (Nagin and Odgers 2010).

We used multinomial logistic regression analysis to examine how social and health-related factors were associated with the selected trajectory groups. In model 1, we adjusted all exposures for age and gender. Model 2 included all social factors (age, gender, marital status, educational level, social support at work, interactional and procedural justice at work, and workplace bullying). In addition to age and gender, model 3 included health-related factors (LTPA, F&V consumption, use of tobacco products, alcohol use, prior SA, and psychological distress). Finally, model 4 included all aforesaid exposures. The results are reported as average marginal effects (AMEs) and their 95% confidence intervals (CIs). AMEs describe the differences between the predictor classes in the predicted probabilities of belonging to each trajectory group, and they have been recommended to be used over odds ratios (ORs) (Landerman et al. 2011; Mood 2010). For example, AMEs produce estimates for each outcome group, contrary to ORs which require a predetermined reference group; thus, AMEs are more tangible to be interpreted and are not as much influenced by the selected reference group, compared to ORs. However, we report also ORs and their 95% CIs from model 1 in the supplementary materials (Tables S3 and S4). We conducted all analyses with Stata version 17 (StataCorp LLC, College Station, TX, USA) and used the traj command for the trajectory analyses (Jones and Nagin 2013).

## Results

A tenth (n = 103) of participants did not have any SA during the 2-year follow-up. More precisely, 11% (n = 115) did not have any short-term SA and 74% (n = 789) did not have any long-term SA (Table S5). Among those who had at least 1 short- or long-term SA day, the median number of days of short- and long-term SA were 13 and 30, respectively, and the median number of spells of short- and long-term SA were 5 and 1, respectively, over the follow-up. Most of the participants were women (81%), had a higher education degree (bachelor’s degree or higher: 63%), and had a partner (61%) (Table 1). Half (52%) of the participants had high LTPA, and 39% consumed F&V daily. A third of the participants had been bullied at work, either previously or currently (29%), used tobacco products (34%), had high alcohol use (32%), and reported high psychological distress (29%).

**Table 1:** Descriptive statistics of the study participants of the Helsinki Health Study in 2017

	N (%)
Total	1060 (100)
Age	
20–29	335 (32)
30–34	350 (33)
35–39	375 (34)
Gender	
Woman	855 (81)
Man	205 (19)
Education	
Upper secondary school/vocational education or lower	395 (37)
Bachelor's degree	356 (34)
Master's degree or higher	309 (29)
Marital status	
Without a partner	413 (39)
Married, cohabiting, or in a registered partnership	647 (61)
Social support at work^a^	
Low	349 (33)
Average	344 (32)
High	367 (35)
Interactional justice at work^b^	
Low	291 (27)
Average	309 (29)
High	460 (43)
Procedural justice at work^b^	
Low	310 (29)
Average	369 (35)
High	381 (36)
Workplace bullying	
Currently	80 (8)
Previously	229 (22)
I do not know	160 (15)
Never	591 (56)
Leisure-time physical activity^c^	
Low	227 (21)
Moderate	279 (26)
High	554 (52)
Fruit and vegetable consumption	
Non-daily	651 (61)
Daily	409 (39)
Use of tobacco products	
Yes	344 (32)
No	716 (68)
Alcohol use^d^	
High	338 (32)
Moderate	722 (68)
Prior sickness absence days one year before the survey	
> 11 days	312 (29)
4–11 days	310 (29)
0–3 days	335 (32)
Employed less than one year	103 (10)
Psychological distress^e^	
High	311 (29)
Moderate	749 (71)

^a^Social support at work was inquired by four items, and the summary score was divided into tertiles

^b^Interactional and procedural justice at work were both assessed with four items, and the summary scores were divided into tertiles

^c^Leisure-time physical activity (LTPA) was assessed by the volume and intensity of LTPA per week, converted into the weekly metabolic equivalent (MET) hours. We classified LTPA into three groups: low (< 14 MET-h/week), moderate (14–29 MET-h/week), and high (≥ 30 MET-h/week)

^d^Alcohol use included questions of weekly units of beer, wine, and spirits, and binge drinking behaviour. Women consuming 7 units and men consuming 14 units of alcohol in a week, and those drinking 6 units of alcohol or more at once at least once a month or more often, were dichotomised into the ‘high alcohol use’ group, and less than that into the ‘moderate alcohol use’ group

^e^Psychological distress was measured by the subscale of emotional wellbeing of the RAND-36 questionnaire, and we dichotomised participants into those with high (emotional wellbeing score 0–50) and those with moderate (emotional wellbeing score > 50 to 68) psychological distress

**Fig. 2:**
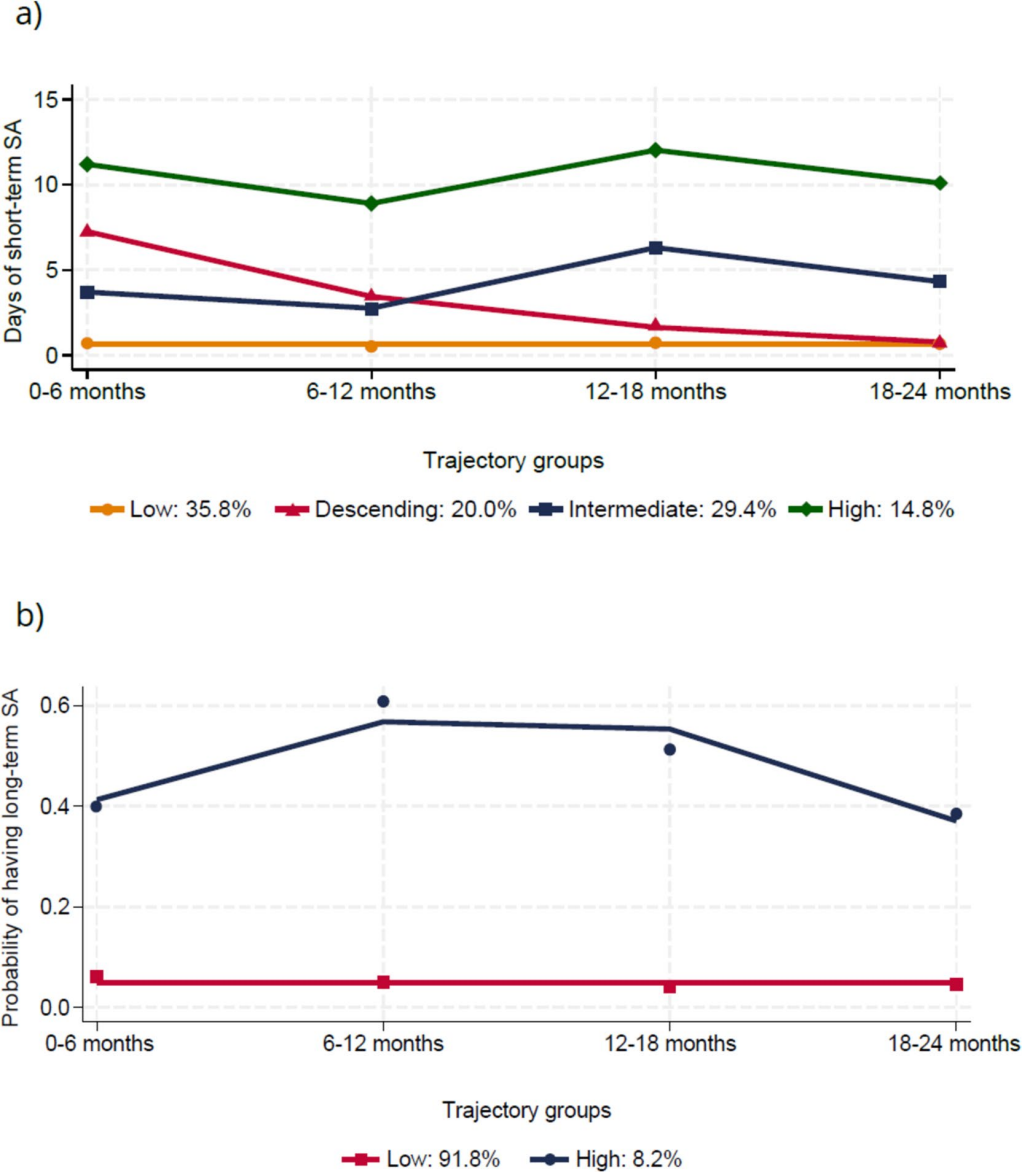
Trajectories of short- (**a**) and long-term (**b**) sickness absence (SA) during a 2-year follow-up among the Helsinki Health Study participants since 2017 (n = 1060). Number of days of short-term SA and probabilities of having long-term SA are modelled. Observed proportions of trajectory group sizes are shown (%)

Figure 2 shows the selected short- and long-term SA trajectories, based on the statistical criteria (see Tables S1 and S2) and the interpretability of the trajectories. We selected four short-term SA trajectories: ‘low’ (n = 379, 36% of participants), ‘descending’ (n = 212, 20%), ‘intermediate’ (n = 312, 29%), and ‘high’ (n = 157, 15%) (Fig. 2a). The ‘low’ trajectory described participants who did not have practically any short-term SA during the follow-up (median number of days: 2 [Table S5]). There were only small changes in the trajectory patterns over the follow-up in the ‘intermediate’ and ‘high’ trajectories as well, and the greatest change was seen in the ‘descending’ trajectory. The median number of short-term SA days over the follow-up was 12 in the ‘descending’ trajectory group and 16 in the ‘descending’ trajectory group, whereas in the ‘high’ trajectory group, it was considerably higher (37 days) (Table S5). For long-term SA, we selected 2 trajectories: ‘low’ (n = 973, 92%), and ‘high’ (n = 87, 8%) (Fig. 2b). Participants assigned to the ‘low’ trajectory group had only a small predicted probability of having any long-term SA during the follow-up (median number of days: 0 [Table S5]), while the participants assigned to the ‘high’ trajectory group had a constantly increased predicted probability of having long-term SA (median number of days: 62 [Table S5]).

Men and those with a master’s degree or higher were assigned more often to the ‘low’ than to the other trajectory groups of short-term SA (Table 2). There were no statistically significant differences in the distributions of other social factors – and neither in lifestyle factors (i.e., LTPA, F&V consumption, and tobacco and alcohol use) – by the trajectory groups. Those with under four days of SA in the previous year and those with moderate psychological distress (compared to high distress) were assigned more often to the ‘low’ than to the other short-term SA trajectory groups. For long-term SA trajectories, participants with a master’s degree or higher and those who had never been bullied at work, who did not use tobacco products, who had a maximum of 11 days of SA in a previous year, and who had moderate psychological distress (compared to high distress) were more often assigned to the ‘low’ than to the ‘high’ trajectory group (Table 3).

**Table 2:** Cross-tabulations of the social and health-related factors by short-term sickness absence (SA) trajectory groups among the Helsinki Health Study participants (n = 1060)

	Trajectory groups of short-term SA, n (%)				Chi-squared test, p-value
	Low, n = 379	Descending, n = 212	Intermediate, n = 312	High, n = 157	
Age					0.190
20–29	104 (27)	67 (32)	103 (33)	61 (39)	
30–34	128 (34)	72 (34)	107 (34)	43 (27)	
35–39	147 (39)	73 (34)	102 (33)	53 (34)	
Gender					0.025
Woman	291 (77)	179 (84)	249 (80)	136 (87)	
Man	88 (23)	33 (16)	63 (20)	21 (13)	
Education					< 0.001
Upper secondary school/vocational education or lower	107 (28)	70 (33)	131 (42)	87 (55)	
Bachelor's degree	123 (32)	72 (34)	113 (36)	48 (31)	
Master's degree or higher	149 (39)	70 (33)	68 (22)	22 (14)	
Marital status					0.120
Without a partner	136 (36)	76 (36)	130 (42)	71 (45)	
Married, cohabiting, or in a registered partnership	243 (64)	136 (64)	182 (58)	86 (55)	
Social support at work^a^					0.102
Low	108 (29)	78 (37)	107 (34)	56 (36)	
Average	117 (31)	70 (33)	104 (33)	53 (34)	
High	154 (41)	64 (30)	101 (32)	48 (31)	
Interactional justice at work^b^					0.178
Low	94 (25)	58 (27)	87 (28)	52 (33)	
Average	108 (29)	74 (35)	86 (28)	41 (26)	
High	177 (47)	80 (38)	139 (45)	64 (41)	
Procedural justice at work^b^					0.839
Low	113 (30)	56 (26)	90 (29)	51 (32)	
Average	128 (34)	75 (35)	109 (35)	57 (36)	
High	138 (36)	81 (38)	113 (36)	49 (31)	
Workplace bullying					0.071
Currently	22 (6)	19 (9)	24 (8)	15 (10)	
Previously	77 (20)	41 (19)	67 (21)	44 (28)	
I do not know	48 (13)	30 (14)	58 (19)	24 (15)	
Never	232 (61)	122 (58)	163 (52)	74 (47)	
Leisure-time physical activity^c^					0.281
Low	68 (18)	56 (26)	67 (21)	36 (23)	
Moderate	111 (29)	48 (23)	79 (25)	41 (26)	
High	200 (53)	108 (51)	166 (53)	80 (51)	
Fruit and vegetable consumption					0.931
Non-daily	235 (62)	126 (59)	193 (62)	97 (62)	
Daily	144 (38)	86 (41)	119 (38)	60 (38)	
Use of tobacco products					0.060
Yes	117 (31)	56 (26)	113 (36)	58 (37)	
No	262 (69)	156 (74)	199 (64)	99 (63)	
Alcohol use^d^					0.722
High	123 (32)	72 (34)	92 (29)	51 (32)	
Moderate	256 (68)	140 (66)	220 (71)	106 (68)	
Prior sickness absence days one year before the survey					< 0.001
> 11 days	45 (12)	62 (29)	109 (35)	96 (61)	
4–11 days	88 (23)	75 (35)	110 (35)	37 (24)	
0–3 days	213 (56)	51 (24)	63 (20)	8 (5)	
Employed less than one year	33 (9)	24 (11)	30 (10)	16 (10)	
Psychological distress^e^					< 0.001
High	82 (22)	79 (37)	101 (32)	49 (31)	
Moderate	297 (78)	133 (63)	211 (68)	108 (69)	

^a^Social support at work was inquired by four items, and the summary score was divided into tertiles

^b^Interactional and procedural justice at work were both assessed with four items, and the summary scores were divided into tertiles

^c^Leisure-time physical activity (LTPA) was assessed by the volume and intensity of LTPA per week, converted into the weekly metabolic equivalent (MET) hours. We classified LTPA into three groups: low (< 14 MET-h/week), moderate (14–29 MET-h/week), and high (≥ 30 MET-h/week)

^d^Alcohol use included questions of weekly units of beer, wine, and spirits, and binge drinking behaviour. Women consuming 7 units and men consuming 14 units of alcohol in a week, and those drinking 6 units of alcohol or more at once at least once a month or more often, were dichotomised into the ‘high alcohol use’ group, and less than that into the ‘moderate alcohol use’ group

^e^Psychological distress was measured by the subscale of emotional wellbeing of the RAND-36 questionnaire, and we dichotomised participants into those with high (emotional wellbeing score 0–50) and those with moderate (emotional wellbeing score > 50 to 68) psychological distress

**Table 3:** Cross-tabulations of the social and health-related factors by long-term sickness absence (SA) trajectory groups among the Helsinki Health Study participants (n = 1,060)

	Trajectory groups of long-term SA, n (%)		Chi-squared test, p-value
	Low, n = 973	High, n = 87	
Age			0.096
20–29	299 (31)	36 (41)	
30–34	328 (34)	22 (25)	
35–39	346 (36)	29 (33)	
Gender			0.278
Woman	781 (80)	74 (85)	
Man	192 (20)	13 (15)	
Education			< 0.001
Upper secondary school/vocational education or lower	345 (35)	50 (57)	
Bachelor's degree	328 (34)	28 (32)	
Master's degree or higher	300 (31)	9 (10)	
Marital status			0.346
Without a partner	375 (39)	38 (44)	
Married, cohabiting, or in a registered partnership	598 (61)	49 (56)	
Social support at work^a^			0.354
Low	317 (33)	32 (37)	
Average	313 (32)	31 (36)	
High	343 (35)	24 (28)	
Interactional justice at work^b^			0.432
Low	262 (27)	29 (33)	
Average	285 (29)	24 (28)	
High	426 (44)	34 (39)	
Procedural justice at work^b^			0.096
Low	276 (28)	34 (39)	
Average	341 (35)	28 (32)	
High	356 (37)	25 (29)	
Workplace bullying			0.023
Currently	70 (7.2)	10 (11)	
Previously	204 (21)	25 (29)	
I do not know	143 (15)	17 (20)	
Never	556 (57)	35 (40)	
Leisure-time physical activity^c^			0.168
Low	202 (21)	25 (29)	
Moderate	261 (27)	18 (21)	
High	510 (52)	44 (51)	
Fruit and vegetable consumption			0.082
Non-daily	590 (61)	61 (70)	
Daily	383 (39)	26 (30)	
Use of tobacco products			0.020
Yes	306 (31)	38 (44)	
No	667 (69)	49 (56)	
Alcohol use^d^			0.369
High	314 (32)	24 (28)	
Moderate	659 (68)	63 (72)	
Prior sickness absence days one year before the survey			< 0.001
> 11 days	254 (26)	58 (67)	
4–11 days	297 (31)	13 (15)	
0–3 days	325 (33)	10 (11)	
Employed less than one year	97 (10)	6 (6.9)	
Psychological distress^e^			< 0.001
High	268 (28)	43 (49)	
Moderate	705 (72)	44 (51)	

^a^Social support at work was inquired by four items, and the summary score was divided into tertiles

^b^Interactional and procedural justice at work were both assessed with four items, and the summary scores were divided into tertiles

^c^Leisure-time physical activity (LTPA) was assessed by the volume and intensity of LTPA per week, converted into the weekly metabolic equivalent (MET) hours. We classified LTPA into three groups: low (< 14 MET-h/week), moderate (14–29 MET-h/week), and high (≥ 30 MET-h/week)

^d^Alcohol use included questions of weekly units of beer, wine, and spirits, and binge drinking behaviour. Women consuming 7 units and men consuming 14 units of alcohol in a week, and those drinking 6 units of alcohol or more at once at least once a month or more often, were dichotomised into the ‘high alcohol use’ group, and less than that into the ‘moderate alcohol use’ group

^e^Psychological distress was measured by the subscale of emotional wellbeing of the RAND-36 questionnaire, and we dichotomised participants into those with high (emotional wellbeing score 0–50) and those with moderate (emotional wellbeing score > 50 to 68) psychological distress

Age- and gender-adjusted regression analyses showed that those aged 35–39 years (AME 0.07, 95% CI 0.00–0.14), men (AME 0.08, 95% CI 0.01–0.16), and those with higher education (bachelor’s degree: AME 0.22, 95% CI 0.15–0.29; master’s degree or higher: AME 0.22; 95% CI 0.15–0.29) had a higher predicted probability of being assigned to the ‘low’ short-term SA trajectory group compared to those aged 20–29 years, women, and those with upper secondary school, vocational education or lower, respectively (Table 4). Additionally, those with high social support at work (AME 0.11, 95% CI 0.04–0.18), who had never been bullied at work (AME 0.12, 95% CI 0.02–0.23), those with moderate LTPA (AME 0.10, 95% CI 0.02–0.18), who had moderate psychological distress (compared to high distress; AME 0.13, 95% CI 0.07–0.13), and, especially, who had fewer SA days during the previous year (e.g., with 0–3 prior SA days: AME 0.49, 95% CI 0.42–0.55) had a higher predicted probability of being assigned to the ‘low’ trajectory group. In contrast, those aged 30–34, men, those with higher education, and those with fewer prior SA had a lower predicted probability of being assigned to the ‘high’ short-term SA trajectory group. Further adjustments for social and health-related factors did not essentially affect these associations, although they slightly attenuated (Tables S6–S8).

**Table 4:** Associations of social and health-related factors with short-term sickness absence (SA) trajectory groups among the Helsinki Health Study participants (n = 1060), adjusted for age and gender

	Trajectory groups of short-term SA AME (95% CI)			
	Low n = 379	Descending n = 212	Intermediate n = 312	High n = 157
Age				
20–29	Ref	Ref	Ref	Ref
30–34	0.05 (– 0.02 to 0.12)	0.01 (– 0.05 to 0.07)	0.00 (– 0.07 to 0.07)	– 0.06 (– 0.11 to 0.00)*
35–39	0.07 (0.00 to 0.14)*	0.00 (– 0.06 to 0.06)	– 0.04 (– 0.11 to 0.03)	– 0.03 (– 0.09 to 0.02)
Gender				
Woman	Ref	Ref	Ref	Ref
Man	0.08 (0.01 to 0.16)*	– 0.05 (– 0.11 to 0.01)	0.02 (– 0.05 to 0.09)	– 0.05 (– 0.10 to 0.00)*
Education				
Upper secondary school/vocational education or lower	Ref	Ref	Ref	Ref
Bachelor's degree	0.08 (0.02 to 0.15)*	0.02 (– 0.03 to 0.08)	– 0.01 (– 0.08 to 0.05)	– 0.09 (– 0.15 to – 0.04)**
Master's degree or higher	0.22 (0.15 to 0.29)***	0.05 (– 0.02 to 0.15)	– 0.11 (– 0.18 to – 0.05)**	– 0.15 (– 0.21 to – 0.10)***
Marital status				
Without a partner	Ref	Ref	Ref	Ref
Married, cohabiting, or in a registered partnership	0.04 (– 0.02 to 0.10)	0.03 (– 0.02 to 0.08)	– 0.03 (– 0.09 to 0.03)	– 0.04 (– 0.08 to 0.01)
Social support at work^a^				
Low	Ref	Ref	Ref	Ref
Average	0.03 (– 0.04 to 0.10)	– 0.02 (– 0.08 to 0.04)	– 0.01 (– 0.07 to 0.06)	– 0.01 (– 0.06 to 0.05)
High	0.11 (0.04 to 0.18)**	– 0.05 (– 0.11 to 0.01)	– 0.03 (– 0.10 to 0.03)	– 0.03 (– 0.08 to 0.02)
Interactional justice at work^b^				
Low	Ref	Ref	Ref	Ref
Average	0.03 (– 0.05 to 0.10)	0.04 (– 0.03 to 0.11)	– 0.02 (– 0.09 to 0.05)	– 0.04 (– 0.10 to 0.01)
High	0.06 (– 0.01 to 0.13)	– 0.02 (– 0.08 to 0.03)	0.00 (– 0.07 to 0.07)	– 0.03 (– 0.09 to 0.02)
Procedural justice at work^b^				
Low	Ref	Ref	Ref	Ref
Average	– 0.01 (– 0.09 to 0.06)	0.02 (– 0.04 to 0.08)	0.00 (– 0.07 to 0.07)	– 0.01 (– 0.07 to 0.04)
High	0.00 (– 0.07 to 0.07)	0.03 (– 0.03 to 0.09)	0.00 (– 0.07 to 0.07)	– 0.03 (– 0.09 to 0.02)
Workplace bullying				
Currently	Ref	Ref	Ref	Ref
Previously	0.07 (– 0.04 to 0.19)	– 0.06 (– 0.17 to 0.04)	– 0.01 (– 0.12 to 0.11)	0.00 (– 0.10 to 0.10)
I do not know	0.03 (– 0.09 to 0.15)	– 0.05 (– 0.16 to 0.06)	0.06 (– 0.07 to 0.18)	– 0.04 (– 0.14 to 0.06)
Never	0.12 (0.02 to 0.23)*	– 0.03 (– 0.13 to 0.07)	– 0.03 (– 0.13 to 0.08)	– 0.06 (– 0.15 to 0.03)
Leisure– time physical activity^c^				
Low	Ref	Ref	Ref	Ref
Moderate	0.10 (0.02 to 0.18)*	– 0.08 (– 0.15 to 0.00)*	– 0.01 (– 0.09 to 0.07)	– 0.01 (– 0.07 to 0.05)
High	0.07 (– 0.01 to 0.14)	– 0.05 (– 0.12 to 0.01)	0.00 (– 0.07 to 0.07)	– 0.01 (– 0.07 to 0.04)
Fruit and vegetable consumption				
Non-daily	Ref	Ref	Ref	Ref
Daily	0.00 (– 0.06 to 0.06)	0.01 (– 0.04 to 0.06)	0.00 (– 0.06 to 0.06)	– 0.01 (– 0.05 to 0.04)
Use of tobacco products				
Yes	Ref	Ref	Ref	Ref
No	0.03 (– 0.03 to 0.09)	0.05 (0.00 to 0.10)*	– 0.05 (– 0.11 to 0.01)	– 0.03 (– 0.08 to 0.01)
Alcohol use^d^				
High	Ref	Ref	Ref	Ref
Moderate	– 0.01 (– 0.07 to 0.06)	– 0.03 (– 0.08 to 0.03)	0.04 (– 0.02 to 0.10)	– 0.01 (– 0.06 to 0.04)
Prior sickness absence days one year before the survey				
> 11 days	Ref	Ref	Ref	Ref
4–11 days	0.13 (0.07 to 0.20)***	0.04 (– 0.02 to 0.11)	0.01 (– 0.07 to 0.08)	– 0.18 (– 0.25 to – 0.12)***
0–3 days	0.49 (0.42 to 0.55)***	– 0.05 (– 0.10 to 0.01)	– 0.16 (– 0.23 to – 0.09)***	– 0.28 (– 0.33 to – 0.23)***
Employed less than one year	0.19 (0.09 to 0.29)***	0.03 (– 0.06 to 0.12)	– 0.07 (– 0.17 to 0.04)	– 0.16 (– 0.24 to – 0.07)***
Psychological distress^e^				
High	Ref	Ref	Ref	Ref
Moderate	0.13 (0.07 to 0.19)***	– 0.08 (– 0.13to – 0.02)**	– 0.04 (– 0.10 to 0.02)	– 0.01 (– 0.06 to 0.04)

Average marginal effects (AMEs) and their 95% confidence intervals (CIs) from multinomial logistic regression are shown

*p < 0.05, **p < 0.01, ***p < 0.001

^a^Social support at work was inquired by four items, and the summary score was divided into tertiles

^b^Interactional and procedural justice at work were both assessed with four items, and the summary scores were divided into tertiles

^c^Leisure-time physical activity (LTPA) was assessed by the volume and intensity of LTPA per week, converted to the weekly metabolic equivalent (MET) hours. We classified LTPA into three groups: low (< 14 MET-h/week), moderate (14–29 MET-h/week), and high (≥ 30 MET-h/week)

^d^Alcohol use included questions of weekly units of beer, wine, and spirits, and binge drinking behaviour. Women consuming 7 units and men consuming 14 units of alcohol in a week, and those drinking 6 units of alcohol or more at once at least once a month or more often, were dichotomised into the ‘high alcohol use’ group, and less than that into the ‘moderate alcohol use’ group

^e^Psychological distress was measured by the subscale of emotional wellbeing of the RAND-36 questionnaire, and we dichotomised participants into those with high (emotional wellbeing score 0–50) and those with emotional (emotional wellbeing score > 50 to 68) psychological distress

Concerning long-term SA trajectory groups, age- and gender-adjusted regression analyses showed that those aged 30–34 years (AME 0.04, 95% CI 0.00–0.08), with higher education (bachelor’s degree: AME 0.05, 95% CI 0.01–0.09; master’s degree or higher: AME 0.10, 95% CI 0.06–0.14), not using tobacco products (AME 0.04, 95% CI 0.00–0.08), with a maximum of 11 prior SA days or employed less than one year (AMEs ranging from 0.13 to 0.15), and with moderate psychological distress (compared to high distress; AME 0.08, 95% CI 0.04–0.12) were more likely to be assigned to the ‘low’ trajectory group (Table 5). Further adjustments for social and health-related factors attenuated the associations only slightly and the associations mostly remained (Table S9). However, moderate alcohol use became statistically significantly associated with the ‘high’ long-term SA trajectory group after these adjustments (Table S9, models 3 and 4).

**Table 5:** Associations of social and health-related factors with long-term sickness absence (SA) trajectory groups among the Helsinki Health Study participants (n = 1,060), adjusted for age and gender

	Trajectory groups of long-term SA AME (95% CI)	
	Low n = 973	High n = 87
Age		
20–29	Ref	Ref
30–34	0.04 (0.00–0.08)*	– 0.04 (– 0.08 to 0.00)*
35–39	0.03 (– 0.02 to 0.07)	– 0.03 (– 0.07 to 0.02)
Gender		
Woman	Ref	Ref
Man	0.02 (– 0.02 to 0.06)	– 0.02 (– 0.06 to 0.02)
Education		
Upper secondary school/vocational education or lower	Ref	Ref
Bachelor's degree	0.05 (0.01–0.09)*	– 0.05 (– 0.09 to – 0.01)*
Master's degree or higher	0.10 (0.06–0.14)***	– 0.10 (– 0.14 to – 0.06)***
Marital status		
Without a partner	Ref	Ref
Married, cohabiting, or in a registered partnership	0.01 (– 0.02 to 0.05)	– 0.01 (– 0.05 to 0.02)
Social support at work^a^		
Low	Ref	Ref
Average	0.00 (– 0.04 to 0.04)	0.00 (– 0.04 to 0.04)
High	0.03 (– 0.01 to 0.06)	– 0.03 (– 0.06 to 0.01)
Interactional justice at work^b^		
Low	Ref	Ref
Average	0.02 (– 0.02 to 0.07)	– 0.02 (– 0.07 to 0.02)
High	0.02 (– 0.02 to 0.07)	– 0.02 (– 0.07 to 0.02)
Procedural justice at work^b^		
Low	Ref	Ref
Average	0.04 (– 0.01 to 0.08)	– 0.04 (– 0.08 to 0.01)
High	0.02 (– 0.01 to 0.06)	– 0.04 (– 0.09 to 0.00)
Workplace bullying		
Currently	Ref	Ref
Previously	0.02 (– 0.06 to 0.10)	– 0.02 (– 0.10 to 0.06)
I do not know	0.02 (– 0.07 to 0.11)	– 0.02 (– 0.11 to 0.07)
Never	0.07 (– 0.01 to 0.14)	– 0.07 (– 0.14 to 0.01)
Leisure-time physical activity^c^		
Low	Ref	Ref
Moderate	0.05 (0.00–0.10)	– 0.05 (– 0.10 to 0.00)
High	0.03 (– 0.02 to 0.08)	– 0.03 (– 0.08 to 0.02)
Fruit and vegetable consumption		
Non-daily	Ref	Ref
Daily	0.03 (0.00–0.06)	– 0.03 (0.06–0.00)
Use of tobacco products		
Yes	Ref	Ref
No	0.04 (0.00–0.08)*	– 0.04 (– 0.08 to 0.00)*
Alcohol use^d^		
High	Ref	Ref
Moderate	– 0.02 (– 0.05 to 0.02)	0.02 (– 0.02 to 0.05)
Prior sickness absence days one year before the survey		
> 11 days	Ref	Ref
4–11 days	0.14 (0.09–0.19)***	– 0.14 (– 0.19 to – 0.09)***
0–3 days	0.15 (0.11–0.20)***	– 0.15 (– 0.20 to – 0.11)***
Employed less than one year	0.13 (0.07–0.19)***	– 0.13 (– 0.19 to – 0.07)***
Psychological distress^e^		
High	Ref	Ref
Moderate	0.08 (0.04–0.12)***	– 0.08 (– 0.12 to – 0.04)***

Average marginal effects (AMEs) and their 95% confidence intervals (CIs) from multinomial logistic regression are shown

*p < 0.05, **p < 0.01, ***p < 0.001

^a^Social support at work was inquired by four items, and the summary score was divided into tertiles

^b^Interactional and procedural justice at work were both assessed with four items, and the summary scores were divided into tertiles

^c^Leisure-time physical activity (LTPA) was assessed by the volume and intensity of LTPA per week, converted into the weekly metabolic equivalent (MET) hours. We classified LTPA into three groups: low (< 14 MET-h/week), moderate (14–29 MET-h/week), and high (≥ 30 MET-h/week)

^d^Alcohol use included questions of weekly units of beer, wine, and spirits, and binge drinking behaviour. Women consuming 7 units and men consuming 14 units of alcohol in a week, and those drinking 6 units of alcohol or more at once at least once a month or more often, were dichotomised into the ‘high alcohol use’ group, and less than that into the ‘moderate alcohol use’ group

^e^Psychological distress was measured by the subscale of emotional wellbeing of the RAND-36 questionnaire, and we dichotomised participants into those with high (emotional wellbeing score 0–50) and those with moderate (emotional wellbeing score > 50 to 68) psychological distress

## Discussion

The main finding of this two-year follow-up study are the observed distinct patterns of SA among young and early midlife employees with psychological distress. Most individuals with distress had no long-term SA spells during the follow-up, but more variations were found concerning short-term SA, illustrating the heterogeneity in SA patterns in this population group. As our secondary finding, we identified some potentially protective social and health-related factors that were associated with the ‘low’ SA trajectory groups. For short-term SA trajectories, these were as follows: having a higher education degree, receiving high social support from a supervisor or colleagues, having never been bullied at the workplace, having moderate LTPA, having fewer prior SA, and having only moderate psychological distress. For long-term SA trajectories, the identified protective factors for SA were as follows: having a higher education degree, not using tobacco products, having fewer prior SA, and having only moderate psychological distress.

To the best of our knowledge, there appears to be no comparable research on both short- and long-term SA trajectories among young and early midlife employees with psychological distress. However, a few studies have investigated employees’ long-term SA trajectories due to diagnosed mental disorders. A previous study by our research group identified 3 SA trajectories due to a diagnosed mental disorder among 50–60-year-old employees over a mean follow-up time of 8.7 years: ‘no’, ‘low’, and ‘high’ (Suur-Uski et al. 2023). A population-based Swedish study examined trajectories of work disability, defined by the combined measure of net months with SA and net months of disability, among 19–30-year-old adults who had a main diagnosis of a common mental disorder (Helgesson et al. 2018). They identified five trajectories: ‘constant low’, ‘fluctuant’, and three increasing groups of work disability over the nine-year follow-up. Compared to the group of participants without mental disorders, participants with mental disorders more often had increasing or high persistent levels of work disability (Helgesson et al. 2018). Another population-based Swedish study of 19–30-year-old adults with symptoms of depression and/or anxiety identified 4 long-term SA trajectories over the 13-year follow-up: ‘high-increasing’, ‘low-increasing’, ‘high-decreasing’, and ‘low-constant’ (Alaie et al. 2023). A Spanish study of 18–28-year-old employees working at privately and publicly held companies, examined trajectories of SA due to diagnosed mental disorders and found the following trajectories during the 3-year follow-up: ‘low stable’, ‘low decreasing’, ‘decreasing’, and ‘increasing’ for women; and ‘low stable’, ‘middle stable’, and ‘high stable’ for men (Ayala-Garcia et al. 2021). Additionally, a Norwegian study of working-age (mean age 41.5 years) patients with common mental disorders identified 3 SA trajectories during the 29.5-month follow-up: ‘resilient’, ‘recovery’, and ‘high risk’ (Sandin et al. 2021).

We found the strongest associations with the ‘low’ short- and long-term SA trajectory groups for having a master’s degree or higher, having fewer prior SA, and having less psychological distress. For instance, participants with master’s degree or higher had 22% higher predicted probability of being assigned to the ‘low’ short-term SA trajectory group than those with upper secondary school, vocational education or lower, and participants with 4–11 prior SA days had 30% higher predicted probability of being assigned to the ‘low’ short-term SA trajectory group than those with over 11 prior SA days. These findings imply that advantageous socioeconomic background and good overall health may operate as protective factors for work ability, even when an individual is living with psychological distress. Our sensitivity analysis, where we further considered the contribution of physical health to the associations, also showed that those with high physical functioning had higher predicted probabilities of being assigned to the ‘low’ short- and long-term SA trajectory groups (Tables S10 and S11). However, adding physical functioning into models 3 and 4 did not affect the current estimates (data not shown). Two previous Swedish studies showed that among individuals with a common mental disorder, depression or anxiety, high educational level was associated with the ‘constant low’ work disability trajectory (Helgesson et al. 2018) or the ‘low-constant’ SA trajectory (Alaie et al. 2023). Additionally, a Spanish study found that among male workers, high income was associated with the ‘low constant’ trajectory of SA due to a mental diagnosis (Ayala-Garcia et al. 2021). However, these three studies could not examine how participants’ socioeconomic status was related to short-term SA trajectories, as we could, but instead, their follow-ups for SA were longer than in our study (i.e., 9, 13, and 3 years, respectively). In contrast, those with low socioeconomic positions, more severe mental health problems, and also with other comorbidities may have an increased risk of SA, especially long-term SA, which has been shown in previous studies (Feeney et al. 1998; Marmot et al. 1995; Sandin et al. 2021; Suur-Uski et al. 2023; van den Berg et al. 2017), but also implied by our study.

Of lifestyle factors, physical activity and, in particular, smoking have had the strongest associations with SA in previous studies (Alavinia et al. 2009; Kanerva et al. 2018; Nielsen et al. 2023; Robroek et al. 2011; van den Berg et al. 2017), which is compatible with our findings. However, lifestyle factors were only moderately (mainly for LTPA and tobacco use) or not at all associated with the SA trajectories. Previous studies also suggest that lifestyle factors do not necessarily have as much impact on the risk of SA and work ability as psychosocial working conditions and common diseases have (Alavinia et al. 2009; van den Berg et al. 2017). In these studies, the associations with the psychosocial working conditions (e.g., job demands, job control, support, and work satisfaction) were stronger especially when the outcome was perceived work ability (van den Berg et al. 2017) or longer spells of SA (Alavinia et al. 2009). However, as for lifestyle factors, we did not find psychosocial working conditions either being associated with the long-term SA trajectories, and high social support and not being bullied at work were associated only with the ‘low’ short-term SA trajectory group. Thus, our findings provide some support that psychosocial working conditions might influence the frequency of short-term SA among young and early midlife employees with psychological distress, but more research is needed to confirm these findings. For example, the use of different measures of psychosocial working conditions, such as those used in van den Berg’s et al. (2017) and Alavinia’s et al. (2009) studies, could reveal whether the associations depend on which aspects of psychosocial working conditions are measured. Nevertheless, our findings imply that ‘less easily modifiable’ factors, such as socioeconomic position and overall health, may have a stronger impact on SA than ‘more easily modifiable’ psychosocial working conditions and lifestyle factors.

Long-term SA spells were generally rare in our study sample. However, while most of the participants had no long-term SA, there was a group of participants who had a constantly high risk of long-term SA. This particular group is important to consider from the perspective of secondary prevention, given that recurring long-term SA has been associated with a higher risk of premature labour market exit due to work disability (Helgesson et al. 2015; Klein et al. 2021). A supplementary analysis showed that the participants who were assigned to the ‘high’ long-term SA trajectory group were also more often assigned to the ‘high’ short-term SA trajectory group compared to the other short-term SA trajectory groups (Table S12). This implies that participants with more short-term SA spells may have an increased risk for long-term SA spells, as previous studies have shown (Harkko et al. 2021; Sumanen et al. 2017). However, most participants (83%) who were assigned to the ‘high’ short-term SA trajectory group were assigned to the ‘low’ long-term SA trajectory group (Table S12). Following these participants’ SA development for a longer time period could show whether these participants with a constantly high number of short-term SA will also have more long-term SA in the future. Meanwhile, this period when only a minority of employees have long-term SA could be a precious time to intervene and support these employees’ mental wellbeing and thus, work ability. A previous quasi-experimental study by our research group showed that seeing an occupational health psychologist reduced SA due to mental disorders (Lahti et al. 2021), which provides one example on how these employees’ well-being could be supported by the employer.

### Strengths and limitations

The strength of this study is that we were able to link the comprehensive questionnaire survey of employees’ social and health-related factors with the employer’s register data on varying lengths of SA. To the best of our knowledge, this is the first study to explore both short- and long-term SA trajectories, and how social and health-related factors are associated with them, among young and early midlife employees with psychological distress. Our study provides information that helps to identify employees with psychological distress who are in a particular need of support and at a high risk of developing work disability. This further helps employers and occupational health professionals to plan targeted secondary prevention actions to maintain and protect employees’ work ability. Using a person-oriented method (i.e., trajectory modelling) instead of traditional variable-oriented methods enabled us to identify heterogeneous pathways in the development of SA among employees with psychological distress, who are often investigated as a homogeneous sub-group.

The limitations of our study should be considered. Firstly, our follow-up time was relatively short, two years, which may be too short to detect change, especially concerning long-term SA. Secondly, as we investigated only employees with psychological distress, there was a relatively small number of cases with long-term SA. This may explain why we were not able to identify more than two long-term SA trajectories. Though the other statistical model selection criteria were fulfilled, we could not find a solution where the OCC criterion would have been met. Thus, although the two-trajectory solution was the best one we found for the long-term SA trajectories, the findings concerning long-term SA trajectories should be interpreted with caution. Thirdly, the survey data were based on self-reports which may cause reporting bias, for example, due to social desirability and recall error (Althubaiti 2016). However, we used validated and commonly used measures, such as emotional wellbeing scale of RAND-36 and Moorman’s measures of organisational justice, which strengthens our analyses. Fourthly, we selected the cut-off score of 68/100 for identifying participants with psychological distress, based on previous literature (Cuijpers et al. 2009; Ten Haven et al. 2024; Yamazaki et al. 2005) and the feasibility of the score for our data and analyses; however, the cut-off scores have slightly varied between the previous studies, which weakens the comparability. The selected cut-off score identifies participants with mild, moderate, or severe psychological distress (Yamazaki et al. 2005), and thus, the findings do not necessarily reflect population groups with severe or clinically significant mental disorders.

Finally, our findings may be influenced by a ‘healthy worker effect’, which means that in comparison to the general population, employed populations are generally healthier (Li and Sung 1999). Additionally, compared to those who did not respond, the survey respondents had more often a higher socioeconomic position, a full-time job, and fewer long-term SA (Lallukka et al. 2020). Thus, it is likely that the findings represent the ‘more advantageous’ group of employees of the City of Helsinki with at least moderate psychological distress, and the social- and health-related associations with SA trajectories might have been stronger among the whole target population. Furthermore, the findings cannot be compared to the general adult population with psychological distress. In Finland, the public sector is strongly female dominated, as were our data, and hence these findings may not apply to men. Due to the small proportion of men, we could not perform gender-stratified analyses. However, our findings support the previous literature in that female employees are in a higher risk of having repeated SA (Alaie et al. 2023; Sandin et al. 2021). In addition, these findings may not be generalisable to other countries with different social security systems and SA policies. The social security system in Finland affects the ways SA is being used, granted, and reported.

## Conclusions

Distinct trajectories of short- and long-term SA can be found among early and mid-career employees with psychological distress. Although only a small group of participants had a high likelihood of having long-term SA spells over the two-year follow-up, repeated short-term SA were much more common among these young and early midlife employees, which may predict future long-term SA later in their careers. Employers could strengthen their employees’ work ability by fostering social support at work, tackling workplace bullying, and endorsing employees’ physical activity, for example. Additionally, the work ability of employees with low socioeconomic positions and those who already have mental health symptoms should especially be supported, for example, via occupational health services. More studies are needed with longer SA follow-ups so that the heterogeneous pathways in the development of SA—and the related factors—among young and early midlife employees with psychological distress could be identified in a more detailed way.

## Supplementary Information

Below is the link to the electronic supplementary material.

Supplementary file1 (DOCX 96 KB)

## Data Availability

The Helsinki Health Study survey data cannot be made publicly available due to strict data protection laws and regulations. The data can only be used for scientific research. More information on the survey data can be requested from the Helsinki Health Study research group (kttl-hhs@helsinki.fi).
