# Supplementary material for: Sickness absence trajectories among young and early midlife employees with psychological distress: the contributions of social and health-related factors in a longitudinal register linkage study
Source: Int Arch Occup Environ Health. 2024 Dec 5;98(1):59–77. doi: 10.1007/s00420-024-02114-7 (PMC11807076; doi:10.1007/s00420-024-02114-7)
Supplement: Supplementary file 1 — Supplementary file1 (DOCX 96 KB) [file 420_2024_2114_MOESM1_ESM.docx]

# SUPPLEMENTARY MATERIALS

**Title:** Sickness absence trajectories among young and early midlife employees with psychological distress: the contributions of social and health-related factors in a longitudinal register linkage study

**Journal:** International Archives of Occupational and Environmental Health

**Authors:** Jatta Salmela*, Noora Amanda Heinonen, Jade Knop, Marianna Virtanen, Pi Fagerlund, Anne Kouvonen, Tea Lallukka

***Corresponding author:** Jatta Salmela; Department of Public Health, University of Helsinki, Finland; [jatta.salmela@helsinki.fi](mailto:jatta.salmela@helsinki.fi)

**Table S1**. Model selection statistics for short-term sickness absence trajectories. The final selected model is bolded.

| **Model** | **Bayesian**  **information**  **criterion**  **(BIC)** | **Trajectory**  **group** | **N** | **Observed**  **proportion**  **(%)** | **Expected**  **proportion**  **(%)** | **Average**  **posterior**  **probability**  **(APP)** | **Odds of**  **correct**  **classification**  **(OCC)** | **Entropy** |
| --- | --- | --- | --- | --- | --- | --- | --- | --- |
| 0 4 | -12792.6 | 1 | 594 | 56.0 | 56.0 | 0.98 | 37.0 | 0.919 |
|  |  | 2 | 466 | 44.0 | 44.0 | 0.97 | 48.3 |  |
| 0 4 4 | -11800.2 | 1 | 381 | 35.9 | 36.4 | 0.96 | 47.3 | 0.900 |
|  |  | 2 | 497 | 46.9 | 46.6 | 0.95 | 20.9 |  |
|  |  | 3 | 182 | 17.2 | 17.1 | 0.95 | 96.2 |  |
| 0 4 4 4 | -11359.3 | 1 | 379 | 35.8 | 34.7 | 0.95 | 32.0 | 0.887 |
|  |  | 2 | 170 | 16.0 | 16.6 | 0.91 | 52.9 |  |
|  |  | 3 | 352 | 33.2 | 33.8 | 0.94 | 30.3 |  |
|  |  | 4 | 159 | 15.0 | 14.9 | 0.95 | 114.6 |  |
| 0 4 4 4 4 | -11079.7 | 1 | 315 | 29.7 | 29.4 | 0.95 | 43.2 | 0.880 |
|  |  | 2 | 260 | 24.5 | 24.7 | 0.89 | 25.3 |  |
|  |  | 3 | 231 | 21.8 | 21.7 | 0.90 | 31.8 |  |
|  |  | 4 | 234 | 22.1 | 22.3 | 0.95 | 69.8 |  |
|  |  | 5 | 20 | 1.9 | 1.9 | 0.97 | 1750.4 |  |
| 0 3 3 3 | -11349.2 | 1 | 376 | 35.5 | 35.1 | 0.95 | 37.0 | 0.876 |
|  |  | 2 | 215 | 20.3 | 20.4 | 0.90 | 36.1 |  |
|  |  | 3 | 313 | 29.5 | 29.7 | 0.92 | 27.2 |  |
|  |  | 4 | 156 | 14.7 | 14.8 | 0.96 | 129.4 |  |
| 0 2 3 3 | -11358.2 | 1 | 377 | 35.6 | 35.2 | 0.95 | 36.1 | 0.877 |
|  |  | 2 | 206 | 19.4 | 19.8 | 0.91 | 39.4 |  |
|  |  | 3 | 319 | 30.1 | 30.1 | 0.92 | 26.1 |  |
|  |  | 4 | 158 | 14.9 | 15.0 | 0.96 | 123.9 |  |
| **0 1 3 3** | **-11345.6** | **1** | **379** | **35.8** | **35.2** | **0.95** | **34.8** | **0.875** |
|  |  | **2** | **212** | **20.0** | **20.2** | **0.90** | **35.9** |  |
|  |  | **3** | **312** | **29.4** | **29.5** | **0.92** | **25.9** |  |
|  |  | **4** | **157** | **14.8** | **15.0** | **0.96** | **136.2** |  |
| 0 0 3 3 | -11584.0 | 1 | 201 | 19.0 | 19.9 | 0.95 | 82.8 | 0.847 |
|  |  | 2 | 344 | 32.5 | 31.6 | 0.88 | 16.3 |  |
|  |  | 3 | 373 | 35.2 | 35.2 | 0.92 | 21.9 |  |
|  |  | 4 | 142 | 13.4 | 13.3 | 0.94 | 109.3 |  |
| 0 1 2 3 | -11429.5 | 1 | 364 | 34.3 | 34.4 | 0.96 | 43.6 | 0.869 |
|  |  | 2 | 242 | 22.8 | 22.5 | 0.89 | 28.3 |  |
|  |  | 3 | 267 | 25.2 | 25.8 | 0.93 | 29.5 |  |
|  |  | 4 | 187 | 17.6 | 17.4 | 0.95 | 81.2 |  |
| 0 1 3 2 | -11371.7 | 1 | 390 | 36.8 | 36.3 | 0.96 | 36.8 | 0.878 |
|  |  | 2 | 216 | 20.4 | 20.2 | 0.89 | 32.0 |  |
|  |  | 3 | 313 | 29.5 | 30.1 | 0.93 | 30.8 |  |
|  |  | 4 | 141 | 13.3 | 13.4 | 0.95 | 123.0 |  |

**Table S2.** Model selection statistics for long-term sickness absence trajectories. The final selected model is bolded.

| **Model** | **Bayesian**  **information**  **criterion**  **(BIC)** | **Trajectory**  **group** | **N** | **Observed**  **proportion**  **(%)** | **Expected**  **proportion**  **(%)** | **Average**  **posterior**  **probability**  **(APP)** | **Odds of**  **correct**  **classification**  **(OCC)** | **Entropy** |
| --- | --- | --- | --- | --- | --- | --- | --- | --- |
| 0 4 | -1245.0 | 1 | 973 | 91.8 | 89.7 | 0.96 | 2.9 | 0.792 |
|  |  | 2 | 87 | 8.2 | 10.4 | 0.84 | 45.3 |  |
| 0 4 4 | -1261.3 | 1 | 229 | 21.6 | 38.0 | 0.85 | 9.1 | 0.531 |
|  |  | 2 | 789 | 74.4 | 58.5 | 0.75 | 2.1 |  |
|  |  | 3 | 42 | 4.0 | 3.4 | 0.70 | 64.9 |  |
| 0 3 | -1245.6 | 1 | 973 | 91.8 | 89.7 | 0.96 | 2.9 | 0.792 |
|  |  | 2 | 87 | 8.2 | 10.4 | 0.84 | 45.3 |  |
| **0 2** | **-1239.3** | **1** | **973** | **91.8** | **89.5** | **0.96** | **2.9** | **0.784** |
|  |  | **2** | **87** | **8.2** | **10.5** | **0.84** | **45.2** |  |
| 0 1 | -1239.5 | 1 | 973 | 91.8 | 87.5 | 0.94 | 2.3 | 0.730 |
|  |  | 2 | 87 | 8.2 | 12.5 | 0.88 | 49.9 |  |

**Table S3.** Associations of social and health-related factors with short-term sickness absence (SA) trajectory groups among the Helsinki Health Study participants (n = 1,060), adjusted for age and gender. Odds ratios (ORs) and their 95% confidence intervals (CIs) from multinomial logistic regression are shown, and the ‘low’ trajectory group (n = 379) is used as a reference group.

|  | **Trajectory groups of short-term SA, OR (95% CI)** | | |
| --- | --- | --- | --- |
|  | **Descending,**  **n = 212** | **Intermediate,**  **n = 312** | **High,**  **n = 157** |
| **Age** |  |  |  |
| 20–29 | ref. | ref. | ref. |
| 30–34 | 0.90 (0.59-1.37) | 0.85 (0.59-1.24) | 0.59 (0.37-0.95)* |
| 35–39 | 0.81 (0.53-1.23) | 0.71 (0.49-1.03) | 0.65 (0.42-1.02) |
| **Gender** |  |  |  |
| Woman | ref. | ref. | ref. |
| Man | 0.62 (0.40-0.97)* | 0.87 (0.60-1.25) | 0.54 (0.32-0.90)* |
| **Education** |  |  |  |
| Upper secondary school/vocational education or lower | ref. | ref. | ref. |
| Bachelor's degree | 0.85 (0.55-1.30) | 0.72 (0.50-1.05) | 0.44 (0.28-0.69)*** |
| Master's degree or higher | 0.68 (0.44-1.05) | 0.36 (0.24-0.53)*** | 0.17 (0.10-0.29)*** |
| **Marital status** |  |  |  |
| Without a partner | ref. | ref. | ref. |
| Married, cohabiting, or in a registered partnership | 1.03 (0.72-1.47) | 0.81 (0.59-1.10) | 0.71 (0.48-1.04) |
| **Social support at work ^a^** |  |  |  |
| Low | ref. | ref. | ref. |
| Average | 0.82 (0.54-1.24) | 0.89 (0.61-1.30) | 0.87 (0.55-1.38) |
| High | 0.58 (0.38-0.87)** | 0.65 (0.45-0.94)* | 0.61 (0.38-0.96)* |
| **Interactional justice at work ^b^** |  |  |  |
| Low | ref. | ref. | ref. |
| Average | 1.11 (0.71-1.73) | 0.86 (0.57-1.29) | 0.69 (0.42-1.13) |
| High | 0.75 (0.49-1.15) | 0.85 (0.59-1.23) | 0.68 (0.44-1.07) |
| **Procedural justice at work ^b^** |  |  |  |
| Low | ref. | ref. | ref. |
| Average | 1.17 (0.76-1.80) | 1.05 (0.72-1.53) | 0.96 (0.61-1.52) |
| High | 1.20 (0.78-1.83) | 1.01 (0.70-1.47) | 0.79 (0.50-1.27) |
| **Workplace bullying** |  |  |  |
| Currently | ref. | ref. | ref. |
| Previously | 0.58 (0.28-1.21) | 0.77 (0.40-1.50) | 0.78 (0.36-1.66) |
| I do not know | 0.70 (0.32-1.50) | 1.06 (0.53-2.13) | 0.70 (0.31-1.59) |
| Never | 0.59 (0.31-1.14) | 0.62 (0.34-1.15) | 0.45 (0.22-0.92)* |
| **Leisure-time physical activity ^c^** |  |  |  |
| Low | ref. | ref. | ref. |
| Moderate | 0.52 (0.32-0.85)** | 0.72 (0.46-1.12) | 0.69 (0.40-1.19) |
| High | 0.64 (0.42-0.98)* | 0.82 (0.55-1.23) | 0.74 (0.46-1.20) |
| **Fruit and vegetable consumption** |  |  |  |
| Non-daily | ref. | ref. | ref. |
| Daily | 1.05 (0.74-1.49) | 1.00 (0.73-1.37) | 0.95 (0.64-1.41) |
| **Use of tobacco products** |  |  |  |
| Yes | ref. | ref. | ref. |
| No | 1.20 (0.82-1.76) | 0.79 (0.57-1.09) | 0.74 (0.49-1.10) |
| **Alcohol use ^d^** |  |  |  |
| High | ref. | ref. | ref. |
| Moderate | 0.89 (0.61-1.29) | 1.19 (0.85-1.66) | 0.97 (0.64-1.46) |
| **Prior sickness absence days one year before the survey** |  |  |  |
| > 11 days | ref. | ref. | ref. |
| 4–11 days | 0.63 (0.38-1.03) | 0.52 (0.33-0.82)** | 0.20 (0.12-0.34)*** |
| 0–3 days | 0.17 (0.11-0.28)*** | 0.12 (0.08-0.19)*** | 0.02 (0.01-0.04)*** |
| Employed less than one year | 0.49 (0.25-0.95)* | 0.35 (0.19-0.64)** | 0.21 (0.10-0.42)*** |
| **Psychological distress ^e^** |  |  |  |
| High | ref. | ref. | ref. |
| Moderate | 0.46 (0.32-0.67)*** | 0.58 (0.41-0.81)** | 0.61 (0.40-0.93)* |

* p < 0.05, ** p < 0.01, *** p < 0.001
^a^ Social support at work was inquired by four items, and the summary score was divided into tertiles.
^b^ Interactional and procedural justice at work were both assessed with four items, and the summary scores were divided into tertiles.
^c^ Leisure-time physical activity (LTPA) was assessed by the volume and intensity of LTPA per week, converted to the weekly metabolic equivalent (MET) hours. We classified LTPA into three groups: low (< 14 MET-h/week), moderate (14–29 MET-h/week), and high (≥ 30 MET-h/week).
^d^ Alcohol use included questions of weekly units of beer, wine, and spirits, and binge drinking behaviour. Women consuming 7 units and men consuming 14 units of alcohol in a week, and those drinking 6 units of alcohol or more at once at least once a month or more often, were dichotomised into the ‘high alcohol use’ group, and less than that into the ‘moderate alcohol use’ group.
^e^ Psychological distress was measured by the subscale of emotional wellbeing of the RAND-36 questionnaire, and we dichotomised participants into those with high (emotional wellbeing score 0–50) and those with emotional (emotional wellbeing score > 50 to 68) psychological distress.

**Table S4.** Associations of social and health-related factors with short-term sickness absence (SA) trajectory groups among the Helsinki Health Study participants (n = 1,060), adjusted for age and gender. Odds ratios (ORs) and their 95% confidence intervals (CIs) from multinomial logistic regression are shown, and the ‘low’ trajectory group (n = 973) is used as a reference group.

|  | **‘High’ long-term SA trajectory group, OR (95% CI)** |
| --- | --- |
| **Age** |  |
| 20–29 | ref. |
| 30–34 | 0.57 (0.33-0.98)* |
| 35–39 | 0.72 (0.43-1.20) |
| **Gender** |  |
| Woman | ref. |
| Man | 0.75 (0.40-1.38) |
| **Education** |  |
| Upper secondary school/vocational education or lower | ref. |
| Bachelor's degree | 0.58 (0.35-0.95)* |
| Master's degree or higher | 0.21 (0.10-0.44)*** |
| **Marital status** |  |
| Without a partner | ref. |
| Married, cohabiting, or in a registered partnership | 0.83 (0.53-1.30) |
| **Social support at work ^a^** |  |
| Low | ref. |
| Average | 0.99 (0.59-1.66) |
| High | 0.70 (0.40-1.22) |
| **Interactional justice at work ^b^** |  |
| Low | ref. |
| Average | 0.77 (0.43-1.35) |
| High | 0.74 (0.44-1.25) |
| **Procedural justice at work ^b^** |  |
| Low | ref. |
| Average | 0.65 (0.38-1.10) |
| High | 0.57 (0.33-0.98)* |
| **Workplace bullying** |  |
| Currently | ref. |
| Previously | 0.82 (0.38-1.81) |
| I do not know | 0.81 (0.35-1.87) |
| Never | 0.43 (0.20-0.92)* |
| **Leisure-time physical activity ^c^** |  |
| Low | ref. |
| Moderate | 0.56 (0.29-1.05) |
| High | 0.69 (0.41-1.16) |
| **Fruit and vegetable consumption** |  |
| Non-daily | ref. |
| Daily | 0.64 (0.39-1.04) |
| **Use of tobacco products** |  |
| Yes | ref. |
| No | 0.59 (0.37-0.93)* |
| **Alcohol use ^d^** |  |
| High | ref. |
| Moderate | 1.28 (0.77-2.11) |
| **Prior sickness absence days one year before the survey** |  |
| > 11 days | ref. |
| 4–11 days | 0.20 (0.11-0.37)*** |
| 0–3 days | 0.14 (0.07-0.27)*** |
| Employed less than one year | 0.26 (0.11-0.62)** |
| **Psychological distress ^e^** |  |
| High | ref. |
| Moderate | 0.39 (0.25-0.61)*** |

* p < 0.05, ** p < 0.01, *** p < 0.001 ^a^ Social support at work was inquired by four items, and the summary score was divided into tertiles.
^b^ Interactional and procedural justice at work were both assessed with four items, and the summary scores were divided into tertiles.
^c^ Leisure-time physical activity (LTPA) was assessed by the volume and intensity of LTPA per week, converted into the weekly metabolic equivalent (MET) hours. We classified LTPA into three groups: low (< 14 MET-h/week), moderate (14–29 MET-h/week), and high (≥ 30 MET-h/week).
^d^ Alcohol use included questions of weekly units of beer, wine, and spirits, and binge drinking behaviour. Women consuming 7 units and men consuming 14 units of alcohol in a week, and those drinking 6 units of alcohol or more at once at least once a month or more often, were dichotomised into the ‘high alcohol use’ group, and less than that into the ‘moderate alcohol use’ group.
^e^ Psychological distress was measured by the subscale of emotional wellbeing of the RAND-36 questionnaire, and we dichotomised participants into those with high (emotional wellbeing score 0–50) and those with moderate (emotional wellbeing score > 50 to 68) psychological distress.

**Table S5.** Median number of sickness absence (SA) days and spells with interquartile ranges (IQR) among all participants and those who had at least one SA day, and by the short- and long-term SA trajectory groups.

|  | **Median number of SA days (IQR)** | **Median number of SA spells (IQR)** |
| --- | --- | --- |
| **All participants (n = 1,060)** |  |  |
| Short-term SA | 11 (4-21) | 5 (2-8) |
| Long-term SA | 0 (0-11) | 0 (0-1) |
| **Participants who had at least one short- (n = 945) or long-term (n = 271) SA day** |  |  |
| Sort-term SA | 13 (6-22) | 5 (3-9) |
| Long-term SA | 30 (15-56) | 1 (1-2) |
| **Short-term SA trajectory groups** |  |  |
| Low (n = 379) | 2 (0-5) | 1 (1-2) |
| Descending (n = 212) | 12 (9-17) | 5 (3-7) |
| Intermediate (n = 312) | 16 (12-22) | 7 (5-9) |
| High (n = 157) | 37 (32-43) | 14 (12-17) |
| **Long-term SA trajectory groups** |  |  |
| Low (n = 973) | 0 (0-0) | 0 (0-0) |
| High (n = 87) | 62 (40-117) | 2 (2-3) |

**Table S6.** Associations of social factors with short-term sickness absence (SA) trajectory groups among the Helsinki Health Study participants (n = 1,060), model 2 ^a^. Average marginal effects (AMEs) and their 95% confidence intervals (CIs) from multinomial logistic regression are shown.

|  | **Trajectory groups of short-term SA, AME (95% CI)** | | | |
| --- | --- | --- | --- | --- |
|  | **Low,**  **n = 379** | **Descending,**  **n = 212** | **Intermediate,**  **n = 312** | **High,**  **n = 157** |
| **Age** |  |  |  |  |
| 20–29 | ref. | ref. | ref. | ref. |
| 30–34 | 0.00 (-0.07-0.07) | 0.00 (-0.07-0.06) | 0.02 (-0.05-0.09) | -0.02 (-0.07-0.03) |
| 35–39 | 0.04 (-0.04-0.11) | -0.01 (-0.08-0.05) | -0.02 (-0.08-0.05) | -0.01 (-0.06-0.05) |
| **Gender** |  |  |  |  |
| Woman | ref. | ref. | ref. | ref. |
| Man | 0.10 (0.03-0.18)** | -0.04 (-0.10-0.01) | 0.01 (-0.06-0.08) | -0.06 (-0.11- -0.02)** |
| **Education** |  |  |  |  |
| Upper secondary school/vocational education or lower | ref. | ref. | ref. | ref. |
| Bachelor's degree | 0.07 (0.00-0.14)* | 0.02 (-0.04-0.08) | 0.00 (-0.07-0.06) | -0.09 (-0.14- -0.03)** |
| Master's degree or higher | 0.20 (0.13-0.28)*** | 0.05 (-0.02-0.11) | -0.10 (-0.17- -0.04)** | -0.15 (-0.20- -0.10)*** |
| **Marital status** |  |  |  |  |
| Without a partner | ref. | ref. | ref. | ref. |
| Married, cohabiting, or in a registered partnership | 0.02 (-0.04-0.08) | 0.03 (-0.02-0.08) | -0.02 (-0.08-0.03) | -0.02 (-0.07-0.02) |
| **Social support at work ^b^** |  |  |  |  |
| Low | ref. | ref. | ref. | ref. |
| Average | 0.02 (-0.05-0.09) | -0.04 (-0.10-0.03) | -0.01 (-0.08-0.07) | 0.02 (-0.03-0.08) |
| High | 0.10 (0.02-0.19)* | -0.07 (-0.14-0.00) | -0.04 (-0.12-0.04) | 0.01 (-0.05-0.07) |
| **Interactional justice at work ^c^** |  |  |  |  |
| Low | ref. | ref. | ref. | ref. |
| Average | 0.00 (-0.09-0.08) | 0.03 (-0.04-0.10) | 0.00 (-0.08-0.08) | -0.03 (-0.09-0.03) |
| High | 0.00 (-0.09-0.08) | -0.03 (-0.10-0.04) | 0.04 (-0.04-0.13) | -0.01 (-0.08-0.06) |
| **Procedural justice at work ^c^** |  |  |  |  |
| Low | ref. | ref. | ref. | ref. |
| Average | -0.05 (-0.12-0.03) | 0.04 (-0.02-0.10) | 0.02 (-0.06-0.09) | -0.01 (-0.07-0.05) |
| High | -0.06 (-0.14-0.02) | 0.07 (0.01-0.14)* | 0.01 (-0.06-0.09) | -0.03 (-0.09-0.03) |
| **Workplace bullying** |  |  |  |  |
| Currently | ref. | ref. | ref. | ref. |
| Previously | 0.07 (-0.04-0.19) | -0.08 (-0.19-0.03) | -0.01 (-0.12-0.11) | 0.00 (-0.09-0.10) |
| I do not know | 0.04 (-0.08-0.17) | -0.06 (-0.18-0.05) | 0.06 (-0.06-0.19) | -0.04 (-0.13-0.05) |
| Never | 0.10 (-0.01-0.21) | -0.05 (-0.15-0.06) | -0.02 (-0.13-0.09) | -0.04 (-0.13-0.05) |

* p < 0.05, ** p < 0.01, *** p < 0.001

^a^ Model 2: age, gender, marital status, educational level, social support at work, interactional and procedural justice at work, and workplace bullying.

^b^ Social support at work was inquired by four items, and the summary score was dichotomised into tertiles so that the lowest tertile indicated high support.

^c^ Interactional and procedural justice at work were both assessed with four items, and the summary scores were dichotomised so that the lowest tertile indicated a high level of justice.

**Table S7.** Associations of health-related factors with short-term sickness absence (SA) trajectory groups among the Helsinki Health Study participants (n = 1,060), model 3 ^a^. Average marginal effects (AMEs) and their 95% confidence intervals (CIs) from multinomial logistic regression are shown.

|  | **Trajectory groups of short-term SA, AME (95% CI)** | | | |
| --- | --- | --- | --- | --- |
|  | **Low,**  **n = 379** | **Descending,**  **n = 212** | **Intermediate,**  **n = 312** | **High,**  **n = 157** |
| **Age** |  |  |  |  |
| 20–29 | ref. | ref. | ref. | ref. |
| 30–34 | 0.04 (-0.02-0.11) | 0.01 (-0.05-0.07) | 0.00 (-0.07-0.07) | -0.05 (-0.10-0.01) |
| 35–39 | 0.06 (-0.01-0.13) | 0.00 (-0.06-0.06) | -0.04 (-0.11-0.03) | -0.02 (-0.07-0.03) |
| **Gender** |  |  |  |  |
| Woman | ref. | ref. | ref. | ref. |
| Man | 0.07 (0.00-0.14) | -0.04 (-0.10-0.02) | 0.03 (-0.05-0.10) | -0.05 (-0.10-0.00) |
| **Leisure-time physical activity ^b^** |  |  |  |  |
| Low | ref. | ref. | ref. | ref. |
| Moderate | 0.09 (0.01-0.16)* | -0.08 (-0.15- -0.01)* | -0.01 (-0.08-0.07) | 0.00 (-0.06-0.06) |
| High | 0.04 (-0.02-0.11) | -0.05 (-0.12-0.01) | 0.01 (-0.06-0.08) | 0.00 (-0.05-0.05) |
| **Fruit and vegetable consumption** |  |  |  |  |
| Non-daily | ref. | ref. | ref. | ref. |
| Daily | -0.01 (-0.07-0.04) | 0.01 (-0.04-0.06) | 0.00 (-0.06-0.06) | 0.00 (-0.04-0.05) |
| **Use of tobacco products** |  |  |  |  |
| Yes | ref. | ref. | ref. | ref. |
| No | 0.02 (-0.03-0.08) | 0.07 (0.02-0.12)** | -0.06 (-0.12-0.00) | -0.03 (-0.08-0.02) |
| **Alcohol use ^c^** |  |  |  |  |
| High | ref. | ref. | ref. | ref. |
| Moderate | -0.03 (-0.09-0.03) | -0.05 (-0.10-0.01) | 0.06 (0.00-0.12)* | 0.02 (-0.03-0.06) |
| **Prior sickness absence days one year before the survey** |  |  |  |  |
| > 11 days | ref. | ref. | ref. | ref. |
| 4–11 days | 0.13 (0.07-0.19)*** | 0.05 (-0.01-0.12) | 0.01 (-0.07-0.08) | -0.19 (-0.25- -0.12)*** |
| 0–3 days | 0.48 (0.41-0.54)*** | -0.03 (-0.09-0.03) | -0.16 (-0.23- -0.09)*** | -0.28 (-0.34-0.23)*** |
| Employed less than one year | -0.19 (-0.09- -0.29)*** | 0.04 (-0.06-0.13) | -0.07 (-0.17-0.04) | -0.16 (-0.24- -0.07)*** |
| **Psychological distress ^d^** |  |  |  |  |
| High | ref. | ref. | ref. | ref. |
| Moderate | 0.09 (0.03-0.14)** | -0.08 (-0.13- -0.02)** | -0.03 (-0.09-0.03) | 0.02 (-0.02-0.06) |

* p < 0.05, ** p < 0.01, *** p < 0.001

^a^ Model 3: age, gender, leisure-time physical activity (LTPA), fruit and vegetable (F&V) consumption, use of tobacco products, alcohol use, prior SA, and psychological distress.

^b^ LTPA was assessed by the volume and intensity of LTPA per week, converted to the weekly metabolic equivalent (MET) hours. We classified LTPA into three groups: low (< 14 MET-h/week), moderate (14–29 MET-h/week), and high (≥ 30 MET-h/week).

^c^ Alcohol use included questions of weekly units of beer, wine, and spirits, and binge drinking behaviour. Women consuming 7 units and men consuming 14 units of alcohol in a week, and those drinking 6 units of alcohol or more at once at least once a month or more often, were dichotomised into the ‘high alcohol use’ group, and less than that into the ‘moderate alcohol use’ group.

^d^ Psychological distress was measured by the subscale of emotional wellbeing of the RAND-36 questionnaire, and we dichotomised participants into those with high (emotional wellbeing score 0–50) and those with moderate (emotional wellbeing score > 50 to 68) psychological distress.

**Table S8.** Associations of social and health-related factors with short-term sickness absence (SA) trajectory groups among the Helsinki Health Study participants (n = 1,060), model 4 ^a^. Average marginal effects (AMEs) and their 95% confidence intervals (CIs) from multinomial logistic regression are shown.

|  | **Trajectory groups of short-term SA, AME (95% CI)** | | | |
| --- | --- | --- | --- | --- |
|  | **Low,**  **n = 379** | **Descending,**  **n = 212** | **Intermediate,**  **n = 312** | **High,**  **n = 157** |
| **Age** |  |  |  |  |
| 20–29 | ref. | ref. | ref. | ref. |
| 30–34 | 0.02 (-0.05-0.08) | 0.00 (-0.07-0.06) | 0.01 (-0.06-0.08) | -0.03 (-0.08-0.03) |
| 35–39 | 0.04 (-0.03-0.11) | -0.01 (-0.07-0.05) | -0.02 (-0.09-0.05) | 0.00 (-0.06-0.05) |
| **Gender** |  |  |  |  |
| Woman | ref. | ref. | ref. | ref. |
| Man | 0.08 (0.00-0.15)* | -0.04 (-0.11-0.02) | 0.02 (-0.05-0.09) | -0.05 (-0.10-0.00)* |
| **Education** |  |  |  |  |
| Upper secondary school/vocational education or lower | ref. | ref. | ref. | ref. |
| Bachelor's degree | 0.02 (-0.04-0.09) | 0.02 (-0.04-0.08) | 0.01 (-0.06-0.08) | -0.05 (-0.11-0.00)* |
| Master's degree or higher | 0.11 (0.03-0.18)** | 0.05 (-0.01-0.12) | -0.07 (-0.14-0.01) | -0.09 (-0.15- -0.04)*** |
| **Marital status** |  |  |  |  |
| Without a partner | ref. | ref. | ref. | ref. |
| Married, cohabiting, or in a registered partnership | 0.00 (-0.06-0.05) | 0.04 (-0.01-0.08) | -0.02 (-0.08-0.03) | -0.01 (-0.05-0.03) |
| **Social support at work ^b^** |  |  |  |  |
| Low | ref. | ref. | ref. | ref. |
| Average | 0.01 (-0.05-0.08) | -0.03 (-0.09-0.04) | 0.00 (-0.07-0.07) | 0.02 (-0.04-0.07) |
| High | 0.09 (0.01-0.17)* | -0.06 (-0.12-0.01) | -0.04 (-0.12-0.04) | 0.00 (-0.06-0.06) |
| **Interactional justice at work ^c^** |  |  |  |  |
| Low | ref. | ref. | ref. | ref. |
| Average | -0.02 (-0.09-0.06) | 0.03 (-0.04-0.10) | 0.00 (-0.07-0.08) | -0.02 (-0.08-0.04) |
| High | -0.02 (-0.10-0.06) | -0.03 (-0.10-0.04) | 0.05 (-0.03-0.13) | 0.00 (-0.07-0.06) |
| **Procedural justice at work ^c^** |  |  |  |  |
| Low | ref. | ref. | ref. | ref. |
| Average | -0.06 (-0.13-0.01) | 0.04 (-0.02-0.10) | 0.02 (-0.05-0.09) | 0.00 (-0.05-0.06) |
| High | -0.07 (-0.15-0.00) | 0.07 (0.00-0.14) | 0.02 (-0.06-0.10) | -0.02 (-0.08-0.04) |
| **Workplace bullying** |  |  |  |  |
| Currently | ref. | ref. | ref. | ref. |
| Previously | 0.05 (-0.06-0.16) | -0.05 (-0.16-0.05) | 0.01 (-0.11-0.12) | 0.01 (-0.07-0.10) |
| I do not know | 0.02 (-0.10-0.13) | -0.06 (-0.16-0.05) | 0.06 (-0.06-0.19) | -0.02 (-0.11-0.06) |
| Never | 0.05 (-0.05-0.16) | -0.02 (-0.12-0.08) | 0.00 (-0.11-0.10) | -0.02 (-0.10-0.06) |
| **Leisure-time physical activity ^d^** |  |  |  |  |
| Low | ref. | ref. | ref. | ref. |
| Moderate | 0.08 (0.00-0.15) | -0.09 (-0.17- -0.02)* | 0.00 (-0.08-0.08) | 0.01 (-0.04-0.07) |
| High | 0.04 (-0.03-0.10) | -0.07 (-0.13-0.00) | 0.02 (-0.05-0.09) | 0.01 (-0.04-0.06) |
| **Fruit and vegetable consumption** |  |  |  |  |
| Non-daily | ref. | ref. | ref. | ref. |
| Daily | -0.02 (-0.07-0.04) | 0.01 (-0.04-0.06) | 0.00 (-0.06-0.06) | 0.01 (-0.04-0.05) |
| **Use of tobacco products** |  |  |  |  |
| Yes | ref. | ref. | ref. | ref. |
| No | 0.01 (-0.06-0.07) | 0.06 (0.00-0.11)* | -0.05 (-0.12-0.01) | -0.01 (-0.06-0.04) |
| **Alcohol use ^e^** |  |  |  |  |
| High | ref. | ref. | ref. | ref. |
| Moderate | -0.03 (-0.09-0.03) | -0.05 (-0.11-0.01) | 0.06 (0.00-0.12)* | 0.01 (-0.03-0.06) |
| **Prior sickness absence days one year before the survey** |  |  |  |  |
| > 11 days | ref. | ref. | ref. | ref. |
| 4–11 days | 0.11 (0.05-0.18)*** | 0.03 (-0.03-0.10) | 0.01 (-0.07-0.09) | -0.15 (-0.22- -0.09)*** |
| 0–3 days | 0.45 (0.38-0.52)*** | -0.05 (-0.11-0.01) | -0.15 (-0.22- -0.08)*** | -0.25 (-0.31- -0.20)*** |
| Employed less than one year | 0.16 (0.06-0.27)** | 0.01 (-0.08-0.10) | -0.06 (-0.16-0.05) | -0.11 (-0.20- -0.02)* |
| **Psychological distress ^f^** |  |  |  |  |
| High | ref. | ref. | ref. | ref. |
| Moderate | 0.08 (0.02-0.14)** | -0.08 (-0.13- -0.02)** | -0.02 (-0.08-0.04) | 0.02 (-0.02-0.06) |

* p < 0.05, ** p < 0.01, *** p < 0.001

^a^ Model 4: age, gender, marital status, educational level, social support at work, interactional and procedural justice at work, workplace bullying, leisure-time physical activity (LTPA), fruit and vegetable (F&V) consumption, use of tobacco products, alcohol use, prior SA, and psychological distress.

^b^ Social support at work was inquired by four items, and the summary score was divided into tertiles.

^c^ Interactional and procedural justice at work were both assessed with four items, and the summary scores were divided into tertiles.

^d^ LTPA was assessed by the volume and intensity of LTPA per week, converted into the weekly metabolic equivalent (MET) hours. We classified LTPA into three groups: low (< 14 MET-h/), moderate (14–29 MET-h/), and high (week ≥ 30 MET-h/).

^e^ Alcohol use included questions of weekly units of beer, wine, and spirits, and binge drinking behaviour. Women consuming 7 units and men consuming 14 units of alcohol in a week, and those drinking 6 units of alcohol or more at once at least once a month or more often, were dichotomised into the ‘high alcohol use’ group, and less than that into the ‘moderate alcohol use’ group.

^f^ Psychological distress was measured by the subscale of emotional wellbeing of the RAND-36 questionnaire, and we dichotomised participants into those with high (emotional wellbeing score 0–50) and those with moderate (emotional wellbeing score > 50 to 68) psychological distress.

**Table S9.** Associations of social and health-related factors with long-term sickness absence (SA) trajectory groups among the Helsinki Health Study participants (n = 1,060), models 2–4 ^a^. Average marginal effects (AMEs) and their 95% confidence intervals (CIs) from multinomial logistic regression are shown.

|  | **Trajectory groups of long-term SA, AME (95% CI)** | | | | | |
| --- | --- | --- | --- | --- | --- | --- |
|  | **Model 2** | | **Model 3** | | **Model 4** | |
|  | **Low,**  **n = 973** | **High,**  **n = 87** | **Low,**  **n = 973** | **High,**  **n = 87** | **Low,**  **n = 973** | **High,**  **n = 87** |
| **Age** |  |  |  |  |  |  |
| 20–29 | ref. | ref. | ref. | ref. | ref. | ref. |
| 30–34 | 0.02 (-0.02-0.06) | -0.02 (-0.06-0.02) | 0.03 (-0.01-0.07) | -0.03 (-0.07-0.01) | 0.03 (-0.01-0.07) | -0.03 (-0.07-0.01) |
| 35–39 | 0.01 (-0.03-0.05) | -0.01 (-0.05-0.03) | 0.02 (-0.02-0.06) | -0.02 (-0.06-0.02) | 0.02 (-0.02-0.06) | -0.02 (-0.06-0.02) |
| **Gender** |  |  |  |  |  |  |
| Woman | ref. | ref. | ref. | ref. | ref. | ref. |
| Man | 0.03 (-0.01-0.06) | -0.03 (-0.06-0.01) | 0.02 (-0.02-0.06) | -0.02 (-0.06-0.02) | 0.02 (-0.02-0.06) | -0.02 (-0.06-0.02) |
| **Education** |  |  |  |  |  |  |
| Upper secondary school/vocational education or lower | ref. | ref. | - | - | ref. | ref. |
| Bachelor's degree | 0.05 (0.00-0.09)* | -0.05 (-0.09-0.00)* | - | - | 0.02 (-0.02-0.06) | -0.02 (-0.06-0.02) |
| Master's degree or higher | 0.09 (0.05-0.13)*** | -0.09 (-0.13-0.05)*** | - | - | 0.05 (0.01-0.09)* | -0.05 (-0.09- -0.01)* |
| **Marital status** |  |  |  |  |  |  |
| Without a partner | ref. | ref. | - | - | ref. | ref. |
| Married, cohabiting, or in a registered partnership | 0.01 (-0.03-0.04) | -0.01 (-0.04-0.03) | - | - | 0.00 (-0.04-0.03) | 0.00 (-0.03-0.04) |
| **Social support at work ^a^** |  |  |  |  |  |  |
| Low | ref. | ref. | - | - | ref. | ref. |
| Average | -0.02 (-0.06-0.02) | 0.02 (-0.02-0.06) |  |  | -0.01 (-0.05-0.03) | 0.01 (0.03-0.05) |
| High | 0.00 (-0.05-0.05) | 0.00 (-0.05-0.05) | - | - | 0.00 (-0.05-0.05) | -0.01 (-0.05-0.05) |
| **Interactional justice at work ^b^** |  |  |  |  |  |  |
| Low | ref. | ref. | - | - | ref. | ref. |
| Average | 0.00 (-0.05-0.04) | 0.00 (-0.04-0.05) |  |  | -0.01 (-0.05-0.03) | 0.01 (0.03-0.05) |
| High | -0.01 (-0.06-0.04) | 0.01 (-0.04-0.06) | - | - | -0.01 (-0.06-0.03) | 0.01 (-0.03-0.06) |
| **Procedural justice at work ^b^** |  |  |  |  |  |  |
| Low | ref. | ref. | - | - | ref. | ref. |
| Average | 0.04 (-0.01-0.08) | -0.04 (0.08-0.01) |  |  | 0.02 (-0.03-0.06) | -0.02 (0.06-0.03) |
| High | 0.04 (-0.01-0.09) | -0.04 (-0.09-0.01) | - | - | 0.02 (-0.03-0.07) | -0.01 (-0.07-0.03) |
| **Workplace bullying** |  |  |  |  |  |  |
| Currently | ref. | ref. | - | - | ref. | ref. |
| Previously | 0.01 (-0.07-0.08) | -0.01 (-0.08-0.07) | - | - | 0.00 (-0.07-0.06) | 0.00 (-0.06-0.07) |
| I do not know | 0.02 (-0.06-0.10) | -0.02 (-0.10-0.06) | - | - | 0.00 (-0.07-0.07) | 0.00 (-0.07-0.07) |
| Never | 0.05 (-0.02-0.12) | -0.05 (-0.12-0.02) | - | - | 0.03 (-0.03-0.09) | -0.03 (-0.09-0.03) |
| **Leisure-time physical activity ^c^** |  |  |  |  |  |  |
| Low | - | - | ref. | ref. | ref. | ref. |
| Moderate | - | - | 0.03 (-0.01-0.08) | -0.03 (-0.08-0.01) | 0.02 (-0.02-0.07) | -0.02 (-0.07-0.02) |
| High | - | - | 0.02 (-0.02-0.06) | -0.02 (-0.06-0.02) | 0.01 (-0.03-0.05) | -0.01 (-0.05-0.03) |
| **Fruit and vegetable consumption** |  |  |  |  |  |  |
| Non-daily | - | - | ref. | ref. | ref. | ref. |
| Daily | - | - | 0.02 (-0.01-0.05) | -0.02 (-0.05-0.01) | 0.02 (-0.02-0.05) | -0.02 (-0.05-0.02) |
| **Use of tobacco products** |  |  |  |  |  |  |
| Yes | - | - | ref. | ref. | ref. | ref. |
| No | - | - | 0.04 (0.01-0.08)* | -0.04 (-0.08- -0.01)* | 0.03 (0.00-0.07) | -0.03 (-0.07-0.00) |
| **Alcohol use ^d^** |  | - |  |  |  |  |
| High | - | - | ref. | ref. | ref. | ref. |
| Moderate | - | - | -0.04 (-0.07- -0.01)* | 0.04 (0.01-0.07)* | -0.04 (-0.07-0.00)* | 0.04 (0.00-0.07)* |
| **Prior sickness absence days one year before the survey** |  |  |  |  |  |  |
| > 11 days | - | - | ref. | ref. | ref. | ref. |
| 4–11 days | - | - | 0.13 (0.08-0.18)*** | -0.13 (-0.18- -0.08)*** | 0.11 (0.07-0.16)*** | -0.11 (-0.16- -0.07)*** |
| 0–3 days | - | - | 0.14 (0.10-0.19)*** | -0.14 (-0.19- -0.10)*** | 0.12 (0.08-0.17)*** | -0.12 (-0.17- -0.08)*** |
| Employed less than one year | - | - | 0.12 (0.06-0.18)*** | -0.12 (-0.18- -0.06)*** | 0.10 (0.04-0.16)** | -0.10 (-0.16- -0.04)** |
| **Psychological distress ^e^** |  |  |  |  |  |  |
| High | - | - | ref. | ref. | ref. | ref. |
| Moderate | - | - | 0.05 (0.02-0.09)** | -0.05 (-0.09- -0.02)** | 0.05 (0.01-0.09)** | -0.05 (-0.09- -0.01)** |

* p < 0.05, ** p < 0.01, *** p < 0.001

^a^ Model 2: age, gender, marital status, educational level, social support at work, interactional and procedural justice at work, and workplace bullying. Model 3: age, gender, leisure-time physical activity (LTPA), fruit and vegetable (F&V) consumption, use of tobacco products, alcohol use, prior SA, and psychological distress. Model 4: age, gender, marital status, educational level, social support at work, interactional and procedural justice at work, workplace bullying, LTPA, F&V consumption, use of tobacco products, alcohol use, prior SA, and psychological distress.

^b^ Social support at work was inquired by four items, and the summary score was divided into tertiles.

^c^ Interactional and procedural justice at work were both assessed with four items, and the summary scores were divided into tertiles.

^d^ LTPA was assessed by the volume and intensity of LTPA per week, converted into the weekly metabolic equivalent (MET) hours. We classified LTPA into three groups: low (< 14 MET-h/week), moderate (14–29 MET-h/week), and high (≥ 30 MET-h/week).

^e^ Alcohol use included questions of weekly units of beer, wine, and spirits, and binge drinking behaviour. Women consuming 7 units and men consuming 14 units of alcohol in a week, and those drinking 6 units of alcohol or more at once at least once a month or more often, were dichotomised into the ‘high alcohol use’ group, and less than that into the ‘moderate alcohol use’ group.

^f^ Psychological distress was measured by the subscale of emotional wellbeing of the RAND-36 questionnaire, and we dichotomised participants into those with high (emotional wellbeing score 0–50) and those with moderate (emotional wellbeing score > 50 to 68) psychological distress.

**Table S10.** Associations of physical functioning ^a^ with short-term sickness absence (SA) trajectory groups among the Helsinki Health Study participants (n = 1,060), adjusted for age and gender. Average marginal effects (AMEs) and their 95% confidence intervals (CIs) from multinomial logistic regression are shown.

|  | **Trajectory groups of short-term SA, AME (95% CI)** | | | |
| --- | --- | --- | --- | --- |
|  | **Low,**  **n = 379** | **Descending,**  **n = 212** | **Intermediate,**  **n = 312** | **High,**  **n = 157** |
| **Physical functioning** |  |  |  |  |
| Low | ref. | ref. | ref. | ref. |
| High | 0.11 (0.04-0.18)** | 0.03 (-0.03-0.09) | -0.07 (-0.14-0.00) | -0.07 (-0.13- -0.01)* |

* p < 0.05, ** p < 0.01, *** p < 0.001

^a^ Physical functioning was measured by the physical functioning subscale (10 items, yielding overall scores of 0–100) of the RAND-36 questionnaire. We dichotomized participants into those with low (the lowest quintile, score ≤ 85) and those with high (four highest quintiles, score >85) physical functioning.

**Table S11.** Associations of physical functioning ^a^ with long-term sickness absence (SA) trajectory groups among the Helsinki Health Study participants (n = 1,060), adjusted for age and gender. Average marginal effects (AMEs) and their 95% confidence intervals (CIs) from multinomial logistic regression are shown.

|  | **Trajectory groups of long-term SA, AME (95% CI)** | |
| --- | --- | --- |
|  | **Low,**  **n = 973** | **High,**  **n = 87** |
| **Physical functioning** |  |  |
| Low | ref. | ref. |
| High | 0.09 (0.04-0.14)** | -0.09 (-0.14- -0.04)** |

* p < 0.05, ** p < 0.01, *** p < 0.001

^a^ Physical functioning was measured by the physical functioning subscale (10 items, yielding overall scores of 0–100) of the RAND-36 questionnaire. We dichotomized participants into those with low (the lowest quintile, score ≤ 85) and those with high (four highest quintiles, score >85) physical functioning.

**Table S12.** Cross-tabulation of the trajectory groups of short- and long-term sickness absence (SA) trajectory groups among the Helsinki Health Study participants (n = 1,060).

| **Long-term SA trajectory groups** | **Short-term SA trajectory groups** | | | | **Chi-squared test,**  **p-value** |
| --- | --- | --- | --- | --- | --- |
|  | Low | Descending | Intermediate | High | <0.001 |
| Low | 374 (99) | 189 (89) | 280 (90) | 130 (83) |  |
| High | 5 (1) | 23 (11) | 32 (10) | 27 (17) |  |
